# Polypyrrole Nanoenzymes as Tumor Microenvironment Modulators to Reprogram Macrophage and Potentiate Immunotherapy

**DOI:** 10.1002/advs.202201703

**Published:** 2022-06-09

**Authors:** Weiwei Zeng, Mian Yu, Ting Chen, Yuanqi Liu, Yunfei Yi, Chenyi Huang, Jia Tang, Hanyue Li, Meitong Ou, Tianqi Wang, Meiying Wu, Lin Mei

**Affiliations:** ^1^ School of Pharmaceutical Sciences (Shenzhen) Shenzhen Campus of Sun Yat‐sen University Shenzhen 518107 China; ^2^ Tianjin Key Laboratory of Biomedical Materials Key Laboratory of Biomaterials and Nanotechnology for Cancer Immunotherapy Institute of Biomedical Engineering Chinese Academy of Medical Sciences and Peking Union Medical College Tianjin 300192 China

**Keywords:** enzyme‐like activities, immunotherapy, macrophage reprogramming, nanoenzymes, NIR I/II biowindow

## Abstract

Nanozyme‐based tumor catalytic therapy has attracted widespread attention in recent years, but its therapeutic outcome is drastically diminished by species of nanozyme, concentration of substrate, pH value, and reaction temperature, etc. Herein, a novel Cu‐doped polypyrrole nanozyme (CuP) with trienzyme‐like activities, including catalase (CAT), glutathione peroxidase (GPx), and peroxidase (POD), is first proposed by a straightforward one‐step procedure, which can specifically promote O_2_ and ·OH elevation but glutathione (GSH) reduction in tumor microenvironment (TME), causing irreversible oxidative stress damage to tumor cells and reversing the redox balance. The PEGylated CuP nanozyme (CuPP) has been demonstrated to efficiently reverse immunosuppressive TME by overcoming tumor hypoxia and re‐educating macrophage from pro‐tumoral M2 to anti‐tumoral M1 phenotype. More importantly, CuPP exhibits hyperthermia‐enhanced enzyme‐mimic catalytic and immunoregulatory activities, which results in intense immune responses and almost complete tumor inhibition by further combining with *α*PD‐L1. This work opens intriguing perspectives not only in enzyme‐catalytic nanomedicine but also in macrophage‐based tumor immunotherapy.

## Graphical abstract

Second near‐infrared light‐activated Cu‐doped polypyrrole nanozyme (CuP) with hyperthermia‐enhanced trienzyme‐like activities, including CAT, POD, and GPx, are elaborately synthesized via a straightforward one‐step procedure for causing irreversible oxidative stress damage to tumor cells and reversing immunosuppressive tumor microenvironment by re‐educating macrophage from pro‐tumoral M2 to anti‐tumoral M1 phenotype.

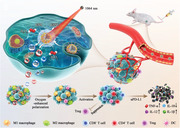

## 1 Introduction

The redox balance between the production and elimination of reactive oxygen species (ROS) plays a pivotal role in maintaining various signaling pathways and normal physiological processes.^[^ ^1^ ^]^ Compared with normal cells, tumor cells harbor high ROS levels due to aberrant proliferation and metabolism, accompanied by the upregulated antioxidant defense systems, especially some ROS‐scavenging enzymes, such as catalase (CAT) and peroxidase (POD), etc.^[^ ^2^ ^]^ The altered redox stability not only contributes to tumor development and progression, but also increases the susceptibility of tumor cells to oxidative damage.^[^ ^3^ ^]^ Therefore, the elevation of ROS in tumor microenvironment (TME) by ROS‐scavenging enzymes or increasing ROS generation presents a new era for tumor therapy.

Natural enzymes suffer from high preparation cost, enzyme activity instability, and harsh reaction condition, which seriously restrain their widespread application.^[^ ^4^ ^]^ As an alternative, artificial enzymes based on nanomaterials named as “nanozymes” with tunable enzyme‐like catalytic activities, high physiological stability, and facile preparation with low cost have been elaborately designed and constructed to exert enzyme function, especially transition metal‐based nanomaterials (e.g., Fe, Cu, Mn, etc.).^[^ ^5^ ^]^ The enzyme‐mimetic catalytic activities of nanozymes are closely related with species of nanozyme, concentration of substrate, pH value, and reaction temperature, etc. Encouragingly, various strategies have been devoted to maximizing the intratumoral enzyme‐like reactions by regulating these abundant influencing factors.^[^ ^6^ ^]^ The emerging Cu‐based nanozymes with polyvalent status (Cu^I^/Cu^II^), identical to the pioneering Fe‐based nanozymes, can specifically catalyze intratumoral H_2_O_2_ and GSH into O_2_ and GSSG by Cu^II^‐mediated CAT‐ and GPx‐like reactions, respectively.^[^ ^7^ ^]^ More importantly, it is much easier for Cu‐based nanozymes to exert Cu^I^‐mediated POD‐like reaction that catalyzes H_2_O_2_ into highly toxic ·OH independent of weakly acidic pH, in which an ≈160‐fold faster reaction rate was realized in contrast to Fe‐based nanozymes due to the low redox potential of Cu^I^/Cu^II^.^[^ ^8^ ^]^ In addition, the higher substrate concentration and/or reaction temperature, the faster enzyme‐like reaction rate can be acquired to enhance therapeutic efficacy of nanozyme.^[^ ^9^ ^]^ On the basis of this mechanism, photothermal therapy (PTT), utilizing absorbed photoenergy to produce a local hyperthermia to noninvasively ablate tumors, has great potential to achieve the maximized cell death by integrating thermal ablation and nanozyme‐based catalytic therapy into one nanoplatform.^[^ ^10^ ^]^ It is thus of great interest to explore a novel Cu‐based nanozyme with hyperthermia‐enhanced catalytic activity for boosting antitumor effects.

Innate immune system, the first line of defense for maintaining body homeostasis, which is closely associated with the aforementioned redox homeostasis, also plays a vital role in tumor growth and metastasis.^[^ ^11^ ^]^ In TME, tumor‐associated macrophages (TAMs) generally switch to an M2‐like phenotype, which display immunosuppressive, anti‐inflammatory, and tumorigenic functions. Conversely, M1‐like macrophages, which are poorly expressed in TME, not only recognize and destroy malignant tumor cells through phagocytosis, but also initiate adaptive immunity by recruiting and activating other immune cells such as cytotoxic T cells through an antigen presentation process.^[^ ^12^ ^]^ Thereby, regulation of tumor immune microenvironment through macrophage reprogramming is a high promising strategy in cancer therapy. Recently, evidences have shown that ROS‐ and/or O_2_‐generating nanoparticles can act as potent immune response initiators and enhancers to re‐educate macrophages from M2 to M1 phenotypes in tumors.^[^ ^13^ ^]^

**Scheme 1:**
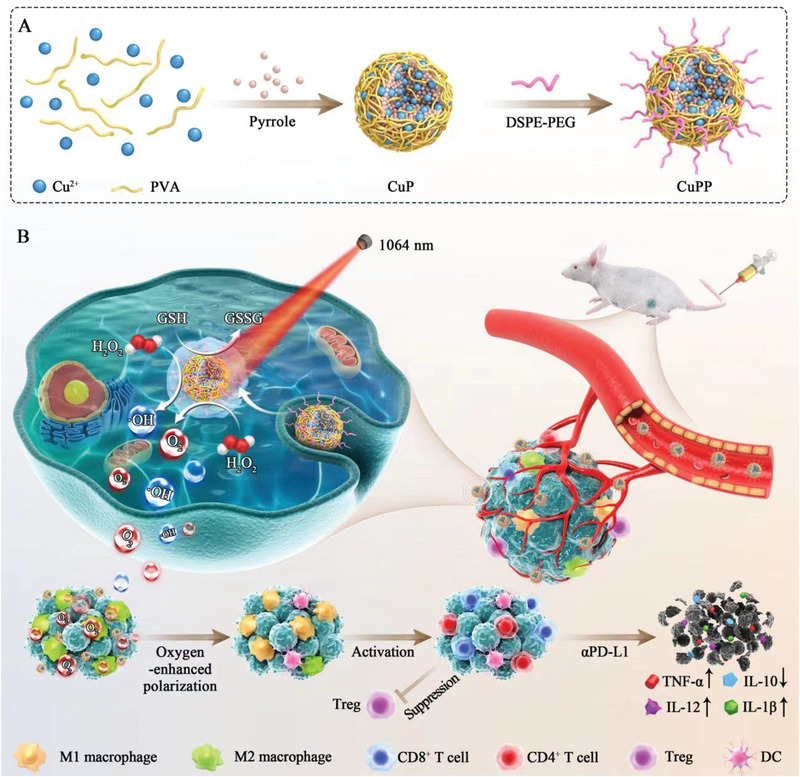
Schematic illustration of the synthetic procedures of CuPP nanozymes with hyperthermia‐enhanced trienzyme‐mimic catalytic activities for synergistic photothermal‐enhanced cancer immunotherapy.

Polypyrrole (PPy) is a well‐known conductive polymer that has been extensively studied in biomedical field with good biocompatibility, controllable size, and tunable photophysical properties.^[^ ^14^ ^]^ The previously reported PPy was mainly synthesized by using FeCl_3_ as oxidizing catalyst, and its biological function was single, mainly manifested in photothermal conversion performance and Fenton catalysis ability.^[^ ^15^ ^]^ Herein, we innovatively developed the preparation method of PPy by replacing FeCl_3_ with CuCl_2_ as oxidizing catalyst via a facile and green one‐step synthesis, and the obtained Cu‐doped PPy (termed as CuP) exhibited remarkable hyperthermia‐enhanced trienzyme‐like activities, including CAT, POD, and GPx, which has been rarely investigated (**Scheme** **1**). After PEGylation, the obtained CuPP could serve as an efficient nanoregulator for alleviating hypoxia and inducing oxidative stress, which therewith reversed immunosuppressive TME by re‐educating macrophage from pro‐tumoral M2 to anti‐tumoral M1 phenotype. The increased ratio of M1/M2 in tumor could initiate adaptive immunity by recruiting and activating immune cells such as cytotoxic T cells. By marriage of CuPP nanozymes and immune checkpoint blocker *α*PD‐L1, the intense immune effect and superb antitumor efficacy could be achieved. Altogether, the designed CuPP nanozymes presented great potential for effective tumor ablation and immune activation.

## 2 Results and Discussion

CuP nanozymes were simply prepared through an in situ chemical oxidative polymerization method using CuCl_2_ rather than commonly used FeCl_3_ as oxidizing catalysts and PVA as stabilizers to initiate polymerization process of pyrrole monomer at room temperature. By regulating PVA amount (from 10 to 100 mg) and meanwhile keeping the other parameters constant, a series of CuP ranging from ≈205 to ≈59 nm was successfully synthesized (Figure S1, Supporting Information). The higher concentration of PVA, the smaller particle size of CuP, which was due to the enhanced steric hindrance effect and solution viscosity with increased amount of PVA.^[^ ^14d^ ^]^ Among those nanoparticles, the obtained CuP with particulate size of ≈100 nm exhibited well‐dispersity and uniform spherical morphology when the amount of PVA added was 30 mg, which were chosen for the following experiments.

**Figure 1:**
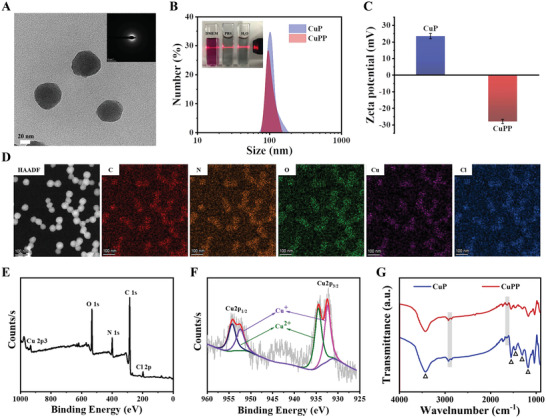
A) HRTEM image of CuPP. Inset shows SAED pattern of CuPP. B) Size distribution of CuP and CuPP measured by DLS. Insets are the digital images of CuPP dispersed in pure water, PBS, and DMEM for one week. C) Zeta potentials of CuP and CuPP. Data represent means ± SD (*n* = 3). D) HAADF‐STEM image of CuPP and the corresponding element‐mapping images. E) XPS spectrum of CuP and F) high‐resolution Cu2p XPS spectrum of CuP spectrum. G) FTIR spectra of CuP and CuPP.

To enhance the potential of CuP nanozymes for biomedical applications, they were further modified by DSPE‐PEG2000 molecules through hydrophobic interaction (denoted as CuPP). As displayed in high‐resolution TEM image and selective area electron diffraction (SAED), the synthesized CuPP exhibited spherical morphology similar to the above CuP and apparent amorphous structure (**Figure** **1**A). In comparison with CuP, the average hydrodynamic diameter of CuPP showed almost no change but the surface zeta potential of CuPP dropped to −27 eV from +23 eV, which were conducive to blood circulation (Figure 1B,C). Moreover, CuPP showed good stability and dispersibility in different physiological media including pure water, phosphate buffer saline (PBS), and Dulbecco's modified Eagle medium (DMEM) for one week (Figure S2, Supporting Information). After that, the chemical composition of CuPP was also a major concern to reflect their physicochemical properties. From the elemental mapping images in Figure 1D, the homogenous and well‐overlapped distribution of Cu, C, N, O, and Cl elements in CuPP demonstrated the successful fabrication of CuPP with Cu‐doped nanostructure. The energy dispersive X‐ray (EDX) spectrum further confirmed the existence of Cu element in the as‐prepared CuP (Figure S3, Supporting Information).

Considering the importance of metal valence state in catalytic process, the typical X‐ray photoelectron spectroscopy (XPS) was also carried out to further reveal their chemical status and metal valence state (Figure 1E,F). From the characteristic peaks existed in Cu2p spectrum, the peaks at 932.3 and 952.4 eV were assigned to Cu^I^, while the peaks at 934.5 and 954.3 eV were associated with Cu^II^. Similarly, the high‐resolution C1s spectrum exhibited four peaks at 284.0, 284.8, 286.3, and 287.8 eV attributed to C*α*, C*β*, C═N/C—N^+^, and C═O of the pyrrole monomer, respectively, and meanwhile the high‐resolution N1s spectrum could be divided into three peaks at 399.6, 401.2, and 402.8 eV assigned to pyrrolic N, C—N^+^, and C═N^+^ of the pyrrole monomer, respectively (Figure S4, Supporting Information). All these results not only demonstrated the intact and typical PPy nanostructure in CuP, but also proved that the doped Cu ions in the framework of CuP were mainly univalent and bivalent that benefited to initiate enzyme‐like catalytic reaction under certain conditions. X‐ray diffraction (XRD) pattern in Figure S5, Supporting Information, revealed that CuP presented the broad hump from 16° to 30° without obvious diffraction peaks, indicating their amorphous nature. Moreover, as shown in Fourier transform infrared (FTIR) spectroscopy (Figure 1G), the characteristic peaks at 3435, 1549, 1457, 1302, and 1170 cm^–1^ belonging to the stretching vibration of N—H, C═C, C—N, C—H, and C—C in traditional PPy framework, respectively, all appeared in the both spectra of CuP and CuPP. Meanwhile, relative to the spectrum of CuP, the absorption peaks at 2918 and 1637 cm^–1^ in CuPP were assigned to the stretching vibration of C—H and C═O in the DSPE‐PEG2000 molecule, respectively, further demonstrating the successful PEGylation on the surface of CuP.

**Figure 2:**
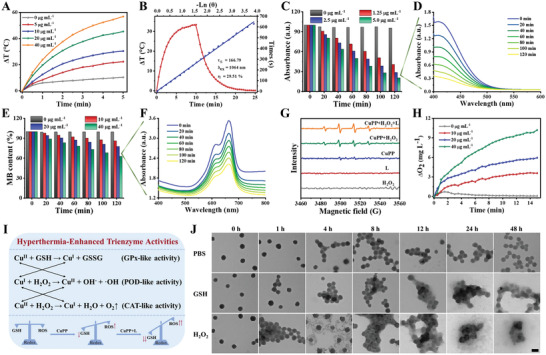
A) Photothermal heating curves of CuPP with different concentrations under irradiation for 5 min by a 1064 nm laser at the power density of 1.0 W cm^–2^. B) Photothermal heating and cooling curves of CuPP under 1064 nm laser, and corresponding linear relationship between time and −ln*θ* from the cooling period. C) The degradation of GSH caused by CuPP with different concentrations (0, 10, 20, and 40 µg mL^–1^). D) The degradation of GSH caused by CuPP at the concentration of 40 µg mL^–1^. E) The degradation of MB caused by the generation of ·OH with different concentrations of CuPP (0, 10, 20, and 40 µg mL^–1^). F) The degradation of MB caused by CuPP at the concentration of 40 µg mL^–1^. G) ESR spectra in varied reaction conditions using 5,5‐dimethyll‐pyrroline *N*‐oxide (DMPO) as a spin trap agent. H) Dissolved O_2_ in different concentrations of CuPP mixed with H_2_O_2_. I) The mechanism of CuPP with trienzyme activities for O_2_ generation and redox homeostasis‐destruction. J) TEM images of CuPP after incubation in pure PBS, GSH, or H_2_O_2_ at different time points. Scale bar: 100 nm.

From the UV–vis–NIR absorption spectra, CuPP nanozymes exhibited broad and strong optical absorbance from 400 to 1300 nm, which was concentration‐dependent (Figure S6A, Supporting Information). Compared with NIR‐I window, the light in NIR‐II window has been identified to allow deeper tissue penetration depth and higher maximum permissible exposure. According to the Lambert–Beer law, the extinction coefficient (*ε*) of CuPP at 808 and 1064 nm were calculated to be 38 and 30 L g^–1^ cm^–1^, respectively, which were prominently higher than that of some classical photothermal agents, such as graphene oxide (3.6 L g^–1^ cm^–1^) (Figure S6B,C, Supporting Information).^[^ ^16^ ^]^ The strong NIR absorption and relatively high *ε* endowed CuPP with great potential to act as efficient photothermal conversion agents under no matter 808 or 1064 nm laser illumination. The photothermal performance of CuPP was next investigated by recording the real‐time temperature of CuPP under varied concentrations and power densities. As shown in **Figure** **2**A, Figures S7A,B and S8A, Supporting Information, CuPP exhibited concentration‐ and power‐dependent temperature elevations. The temperature change (Δ*T*) of CuPP could increase by 55.9 and 56.9 °C after 808 and 1064 nm laser irradiation within 5 min under a relatively low concentration (40 µg mL^–1^) and power density (1.0 W cm^–2^), respectively. In contrast, insignificant temperature increase occurred in pure water irradiated under the same condition, demonstrating that CuPP could efficiently and rapidly convert NIR I/II light into thermal energy. Based on the results of time constant for heat transfer, the photothermal conversion efficiency (*η*) of CuPP were calculated to be 27.86 % at 808 nm and 29.51 % at 1064 nm, respectively, (Figure 2B, and Figure S7D, Supporting Information). More importantly, CuPP remained remarkable photostability over five successive cycles of heating/cooling processes upon irradiation of 808 or 1064 nm laser (Figures S7C and S8B, Supporting Information). Meanwhile, the absorption spectra of CuPP showed scarcely any decline before and after exposure to 808 or 1064 nm laser for 30 min at the same condition (Figure S9, Supporting Information), indicating the excellent photothermal conversion stability of CuPP.

Transition metal with multivalence states has been demonstrated to show fascinating enzyme‐catalytic activities for tumor‐specific therapy, such as Fe^II^/Fe^III^, Cu^I^/Cu^II^, and Co^V^/Co^VI^ (e.g., GSH depletion via GPx‐like activity, O_2_ generation via CAT‐like activity, and ·OH generation via POD‐like activity).^[^ ^17^ ^]^ The disruption of redox homeostasis in TME would definitely induce deleterious effects to tumor cells. Benefitting from the abundant Cu^I^/Cu^II^ active sites in CuPP mentioned above, we initially evaluated the GPx‐like activity of CuPP by using 5,5‐dithiobis‐(2‐nitrobenzoic acid) (DTNB) as the specific probe. Rapid and sustained concentration‐dependent consumption of GSH was observed in CuPP (Figure 2C,D; Figure S10, Supporting Information), which was attributed to the occurred redox reaction between Cu^II^ active sites and GSH, indicating the significant reduction in oxidation resistance property after CuPP treatment. More interestingly, much obvious and faster depletion of GSH could be achieved with reaction temperature increasing (Figure S11, Supporting Information), verifying that the GPx‐like enzyme‐catalytic activity of CuPP could be further accelerated by high temperature (such as hyperthermia induced by PTT). Importantly, Cu^I^ derived from the active centers of CuPP nanozymes and the reaction between Cu^II^ and GSH could catalyze overexpressed H_2_O_2_ in TME into highly toxicity ·OH and Cu^II^ via POD‐like enzyme‐catalytic reaction. By co‐incubating H_2_O_2_ with various concentrations of CuPP at room temperature and using methylene blue (MB) as a probe to detect ·OH generation at the same time, the progressively increased degradation of MB with elevated concentration of CuPP nanozymes could be observed, which broke through pH limitation of ·OH generation compared with Fe‐doped polypyrrole (Figure 2E,F; Figures S12,S13, Supporting Information). In detail, the degradable percentage of MB could reach its maximum at CuPP concentration of 40 µg mL^–1^ within 120 min, while no obvious decrease of MB content was observed in control group without nanozymes. Comparably, when the reaction temperature was rose to 50 °C, the MB degradable percentage caused by CuPP (40 µg mL^–1^) increased to as high as ≈68 % within shorter time (30 min), ascribing to the accelerated POD‐like activity at relatively high temperature (Figure S14, Supporting Information). Encouragingly, we further investigated the MB degradation as well as ·OH generation of CuPP coupling with 1064 nm laser, and the results in Figure S15, Supporting Information, displayed that PTT‐induced hyperthermia would prominently improve the generation of ·OH in contrast to single CuPP or single 1064 nm laser irradiation. Meanwhile, the electron spin resonance (ESR) measurements presented the similar outcomes with MB degradation experiments, further confirming the hyperthermia‐enhanced POD‐like activity and ·OH generation by coupling CuPP with laser (Figure 2G). In addition, the CAT‐like activity of CuPP nanozymes was further investigated by which the O_2_ was generated through decomposition of H_2_O_2_. As shown in Figure 2H and Figures S16,S17, Supporting Information, concentration‐dependent O_2_ production could be achieved when incubating different concentrations of CuPP with equal H_2_O_2_, which would be further enhanced by high temperature or laser‐activated hyperthermia. In general, the self‐circulating system between Cu^I^ and Cu^II^ active centers in CuPP nanozymes was beneficial to persistently and powerfully generate O_2_ and ·OH and simultaneously consume GSH, leading to alleviation of tumor hypoxia as well as occurrence of oxidative stress (Figure 2I).

In addition to evaluating the hyperthermia‐enhanced trienzyme‐catalytic performance and mechanism of CuPP, their biodegradable behaviors in simulated TME were also the crucial factors in future potential clinical application. As shown in Figure 2J, the framework of CuPP still remained relatively intact after incubation with pure PBS even for 48 h, illustrating their good physiological stability. However, when dispersing in PBS containing GSH, CuPP exhibited the apparent structural rupture at 12 h. Moreover, the most serious degradation behavior was presented after incubating CuPP with PBS containing H_2_O_2_, with almost complete fragmentation at 48 h, indicating their specific biodegradability in TME and relatively biosafe in normal tissues. Besides, such GSH‐ and H_2_O_2_‐responsive degradation behaviors of CuPP further confirmed the trienzyme‐catalytic activities due to the presence of Cu^I^/Cu^II^ mixed valance states.

**Figure 3:**
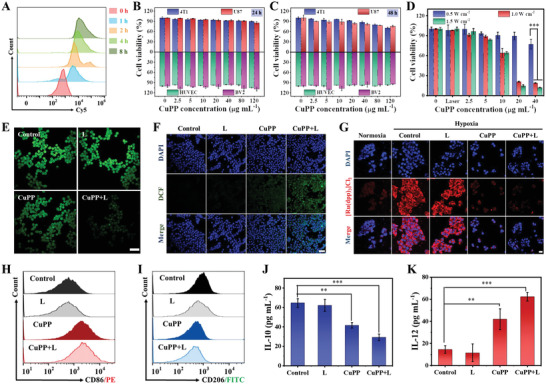
A) Flow cytometry analysis of 4T1 cells incubated with Cy5‐labelled CuPP for different times. Relative viabilities of 4T1, U87, HUVEC, and BV2 cells treated with different concentrations of CuPP (0, 2.5, 5, 10, 20, 40, 80, and 120 µg mL^–1^) for B) 24 or C) 48 h. Data represent means ± SD (*n* = 3). D) Relative viabilities of 4T1 cells after being treated with CuPP under a 1064 nm laser irradiation with different power densities (0.5, 1.0, and 1.5 W cm^–2^) for 5 min. Data represent means ± SD (*n* = 3). E) CLSM images of intracellular GSH levels in 4T1 cells after different treatments. Scale bar: 50 µm. F) CLSM images of intracellular **·**OH in 4T1 cells after different treatments. Scale bar: 100 µm. G) CLSM images of intracellular O_2_ in 4T1 cells after different treatments. Scale bar: 20 µm. Flow cytometric analysis of expressions of H) CD86 (M1 macrophage marker) and I) CD206 (M2 macrophage marker) after different treatments. Secretion levels of J) IL‐10 and K) IL‐12 in the supernatant after different treatments. Data represent means ± SD (*n* = 3). Statistical significance was calculated by one‐way ANOVA analysis. *p < 0.05; **p < 0.01; ***p < 0.001.

Encouraged by the excellent photothermal conversion and hyperthermia‐enhanced catalytic performance of CuPP nanozymes, the TME‐modulating capacity and combined antitumor effect of CuPP were further investigated at the cellular level with or without 1064 nm laser irradiation. At first, the cellular internalization behavior of CuPP labelled with Cy5 dye was investigated by confocal laser scanning microscopy (CLSM) and flow cytometry. The result revealed that CuPP exhibited time‐dependent cellular uptake, as evidenced by the gradually enhanced red fluorescence via CLSM as well as their corresponding quantitative analysis via flow cytometry (**Figure** **3**A; Figure S18, Supporting Information). Whereafter, the cytotoxicity of CuPP was tested in different cell lines after 24 or 48 h incubation by Cell Counting Kit‐8 (CCK‐8) assay. For normal cells (HUVEC and BV2 cells), CuPP revealed negligible cytotoxicity even at a relatively high concentration of 120 µg mL^–1^, which might be attributed to the low H_2_O_2_ concentration and well‐established antioxidant mechanisms in normal cells. In sharp contrast, after incubating CuPP with cancer cells, the viabilities of U87 and 4T1 cells decreased slightly at 24 h incubation and further declined when incubation time extended to 48 h owing to their enzyme‐mimic catalytic activities and overexpressed H_2_O_2_ in tumor cells, illustrating the high biocompatibility of CuPP toward normal cells but specific killing effect to cancer cells (Figure 3B,C). Impressively, the 4T1 cell viabilities dropped significantly after receiving 1064 nm laser irradiation owing to the combined therapeutic efficiency of hyperthermia and enhanced catalytic activity of nanozymes under laser irradiation (Figure 3D). The hemolytic property of CuPP was also conducted and it was found that the hemolytic rate of CuPP to erythrocytes (RBCs) was extremely low at all test concentrations, suggesting the good hemocompatibility and biocompatibility of CuPP (Figure S19, Supporting Information).

Next, we evaluated the trienzyme‐mimic catalytic properties of CuPP for modulating TME (GSH depletion, ·OH and O_2_ generation) at the cellular level. At first, the GSH depletion effect of CuPP in 4T1 cells was investigated by employing the GSH assay kit. Compared to the control group, the green fluorescence in cells treated with CuPP mildly declined but drastically decreased after combination with 1064 nm laser irradiation, demonstrating the superior hyperthermia‐enhanced GSH depletion ability via GPx‐like enzyme activity (Figure 3E; Figure S20, Supporting Information). In the meantime, the ROS generation level in 4T1 cells was determined by the 2′,7′‐dichlorofluorescein diacetate (DCFH‐DA) staining, which could be oxidized by ROS to present green fluorescence. As shown in Figure 3F, negligible ROS fluorescence signal was observed in both control and single laser irradiation groups, while the green fluorescence was enhanced in CuPP group owing to POD‐ and GPx‐like enzyme‐catalytic reactions. Notably, the strongest ROS fluorescence signal was observed in cells treated with CuPP plus laser irradiation, which ascribed to hyperthermia‐enhanced trienzyme‐like activities. The intracellular O_2_ generation capacity was further investigated by incubating 4T1 cells with pure DMEM or CuPP for 12 h under hypoxic condition. The strong red fluorescence signal in both control and single laser irradiation groups indicated the severe hypoxic microenvironment (Figure 3G). Comparatively, the red fluorescence signal was reduced after CuPP treatment and further weakened after laser irradiation, suggesting that CuPP was able to produce sufficient O_2_ for alleviating hypoxia and modulating TME due to their CAT‐like enzyme activity.

Although CuPP manifested safe and high‐efficiency antitumor activity, tumors would still recur and metastasize due to the local residual cancer cells. Therefore, it is of great importance to stimulate the patient's innate immune system to recognize and attack cancer cells. Fortunately, it is well demonstrated that the elevated ROS and/or O_2_ can act as potent immune response initiator and enhancers to polarize macrophage from pro‐tumoral M2 phenotype toward anti‐tumoral M1 phenotype in tumors.^[^ ^12^ ^]^ Therefore, we then explored the potential of CuPP nanozymes in modulating the immunosuppressive TME through inducing macrophage reprogramming. As shown in Figure 3H,I, in contrast to control group, the ratio of M1 macrophages after CuPP treatment was significantly increased while that of M2 macrophages was obviously decreased, demonstrating that CuPP could successfully induce macrophage polarization from M2 phenotype to M1 phenotype. Meanwhile, the supernatant was gathered to detect the cytokines of IL‐12 (secreted by M1 macrophages) and IL‐10 (secreted by M2 macrophages). As shown in Figure 3J,K, the polarization of TAMs from M2 phenotype to M1 phenotype was further verified by the upregulation of IL‐12 from 14 pg mL^–1^ in control group to 62 pg mL^–1^ in CuPP + L group and the downregulation of IL‐10 from 64 pg mL^–1^ in control group to 29 pg mL^–1^ in CuPP + L group. These results indicated that CuPP plus 1064 nm laser irradiation could efficiently reverse immunosuppressive TME by re‐educating M2 macrophages to M1 macrophages.

**Figure 4:**
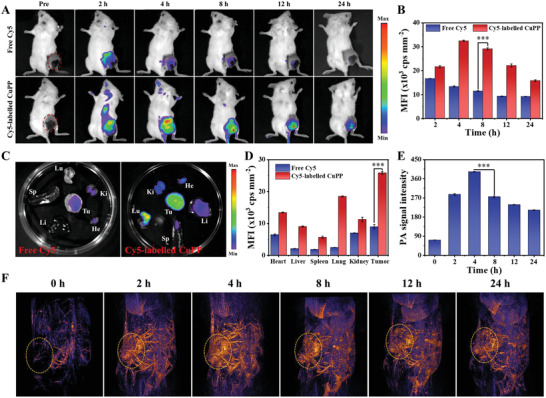
A) In vivo fluorescence images of 4T1 tumor‐bearing mice at different time points after i.v. injection of free Cy5 or Cy5‐labelled CuPP. B) Quantitative analysis of fluorescence signal intensity in tumor regions. Data represent means ± SD (*n* = 3). C) Ex vivo fluorescence images of major organs (heart, liver, spleen, lung, and kidney) and tumor dissected from the mice after 24 h injection of free Cy5 or Cy5‐labelled CuPP. D) The quantified relative fluorescence intensity of major organs and tumors. Data represent means ± SD (*n* = 3). F) In vivo PA imaging and E) corresponding PA signal intensity of tumor tissue before and after i.v. administration of CuPP under 1064 nm laser irradiation at different time points (0, 2, 4, 8, 12, and 24 h). Data represent means ± SD (*n* = 3). Statistical significance was calculated by one‐way ANOVA analysis. *p < 0.05; **p < 0.01; ***p < 0.001.

Encouraged by the outstanding antitumor effect and feasible reversal of immunosuppressive TME in vitro, we next investigated the in vivo biological behavior of CuPP nanozymes on 4T1 tumors. The in vivo biodistribution of Cy5‐labelled CuPP in 4T1 tumor‐bearing mice was first explored and the results displayed that Cy5‐labelled CuPP could rapidly accumulate in tumor regions after intravenous (i.v.) administration and reached its maximum at 4 h post‐injection (**Figure** **4**A,B). Although the accumulation of CuPP in tumor sites decreased slightly with time, it still had high tumor retention at 24 h after i.v. injection. In contrast, free Cy5 revealed fairly low accumulation and retention in tumor regions. The ex vivo fluorescent images further proved that Cy5‐labelled CuPP were mainly accumulated in tumor tissue rather than other major organs, including heart, liver, spleen, lung, and kidney (Figure 4C,D). Subsequently, benefited from the strong and broad NIR I/II absorption and excellent photothermal conversion performance of CuPP nanozymes, we further investigated their PA imaging performance in vitro and in vivo. The PA signal intensity was gradually enhanced with the elevated concentrations of CuPP with exposure to 1064 nm laser (Figure S21, Supporting Information), suggesting that CuPP could be promising contrast agents for in vivo PA imaging in NIR II window. As shown in Figure 4E,F, the prominent contrast enhancement of PA images in the tumor area was confirmed by i.v. injection of CuPP, with an increase of ≈5.4‐fold when exposed to 1064 nm laser irradiation at 4 h. Furthermore, infrared thermal imaging was also employed to monitor the real‐time temperature variation of tumor area during the irradiation (Figure S22, Supporting Information). The temperature of tumor area rapidly increased by 23.8 °C after i.v. injection of CuPP for 300 s, while that in control group had almost no change under the same irradiation conditions, proving the good photothermal conversion performance of CuPP in vivo. These above results strongly evidenced the high accumulation of CuPP in tumor sites, as well as their high potential for further in vivo cancer treatment.

**Figure 5:**
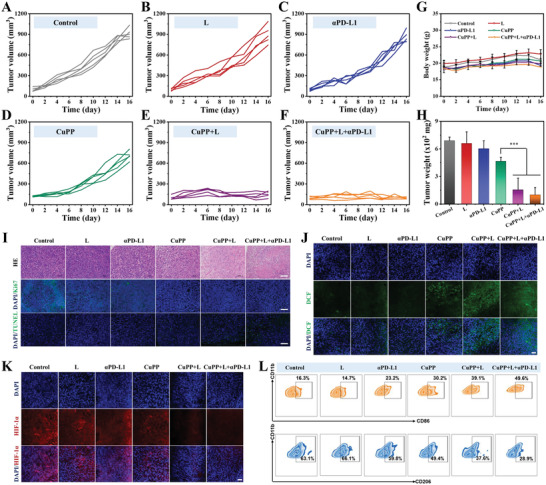
A–F) Individual tumor growth curves of mice in different groups. G) Body weight curves of mice after different treatments. Data represent means ± SD (*n* = 5). H) Tumor weight in different groups after 16 days of treatment. Data represent means ± SD (*n* = 5). Statistical significance was calculated by one‐way ANOVA analysis. *p < 0.05; **p < 0.01; ***p < 0.001. I) HE staining, TUNEL staining, and Ki67 immunofluorescence staining for pathological changes and cellular proliferation in tumor tissues collected from different groups. Scale bar: 100 µm. J) In vivo ROS detection in tumor sections by dichlorodihydrofluorescein via fluorescence microscopy. Scale bar: 50 µm. K) In vivo O_2_ generation in tumor sections by HIF‐1*α* antibody via fluorescence microscopy. Scale bar: 50 µm. L) The percentage of M1 and M2 macrophages in tumors collected from different groups.

To further explore the in vivo therapeutic effect of CuPP nanozymes, 4T1 tumor‐bearing mice were randomly divided into six groups (*n* = 5) and received different treatments as follows: PBS (Control), laser irradiation (L), *α*PD‐L1, CuPP, CuPP + L, and CuPP + L + *α*PD‐L1. The tumor volumes and body weights of 4T1 tumor‐bearing mice in six groups were monitored every 2 days to assess the antitumor ability of CuPP. It was found that both L and *α*PD‐L1 groups showed remarkable tumor growth, similar to control group, illustrating that those groups had almost no inhibitory effects on tumor growth. Comparatively, the mice in CuPP group exhibited certain antitumor outcome within 16 days owing to their trienzyme‐mimic catalytic performance. In sharp contrast, after exposing to 1064 nm laser, almost complete elimination of tumors was observed in CuPP + L group, indicating the remarkably enhanced therapeutic efficacy by synergizing PTT and hyperthermia‐enhanced enzyme catalytic activities. Besides, stronger tumor inhibitory effect could be realized for CuPP + L after combing immune checkpoint blocker (*α*PD‐L1) (**Figure** **5**A–F,H; Figures S23,S24, Supporting Information). Furthermore, the body weights of all mice, as an indicator of systemic toxicity, exhibited no obvious fluctuations during the treatment periods, implying the high biological safety of CuPP nanoenzymes (Figure 5G). The pathological and microenvironmental changes in excised tumors were further investigated. Hematoxylin and eosin (HE) staining of tumors in Figure 5I showed a significantly decreased percentage of nucleus stained with blue in CuPP + L group, slightly higher than that in CuPP + L + *α*PD‐L1 group, implying the serious tumor destruction in mice from the both groups. The proliferative activities of tumor cells measured by Ki67 antibody staining further demonstrated CuPP presented strong suppressive capability against tumor growth under the laser irradiation (Figure S25A, Supporting Information). In the meantime, TdT‐mediated dUTP‐biotin nick end labeling (TUNEL) staining assay also revealed highly significant tumor cell apoptosis or necrosis in CuPP + L and CuPP + L + *α*PD‐L1 groups compared with that in control group (Figure S25B, Supporting Information). The strongest green fluorescence detected in CuPP + L + *α*PD‐L1 group indicated the highest amount of ROS production, which further confirmed the best therapeutic effect of CuPP plus laser irradiation and immune checkpoint blocker (Figure 5J; Figure S25C, Supporting Information). Impressively, the tumor hypoxic levels could be notably relieved after CuPP treatment and further strengthened after addition of 1064 nm laser due to their superior CAT‐like enzyme activity and hyperthermia‐enhanced effect, which contributed to promote the macrophage reprogramming (Figure 5K; Figure S25D, Supporting Information).

**Figure 6:**
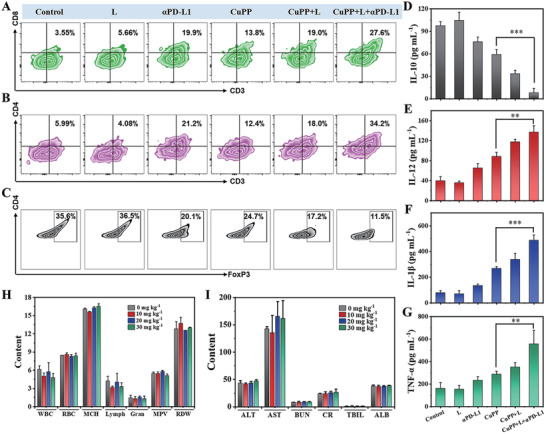
The percentage of A) CD8^+^ T, B) CD4^+^ T, and C) Treg cells in tumors after different treatments. ELISA analysis results of D) IL‐10, E) IL‐12, F) IL‐1*β*, and G) TNF‐*α* in lymph nodes after different treatments. Data represent means ± SD (*n* = 3). H) Blood routine analysis and I) blood biochemical analysis of the mice injected with different CuPP doses of 0, 10, 20, and 30 mg kg^–1^ on day 16. Data represent means ± SD (*n* = 3). Statistical significance was calculated by one‐way ANOVA analysis. *p < 0.05; **p < 0.01; ***p < 0.001.

To get deep insight into immune microenvironment regulation induced by CuPP with or without the help of *α*PD‐L1, the distributed proportion of immune cells (including macrophages, CD4^+^ T cells, CD8^+^ T cells, and Treg cells) in tumor and the secretion of immune cytokines (including IL‐10, IL‐12, IL‐1*β*, and TNF‐*α*) in lymph nodes were further analyzed. As shown in Figure 5L, compared with control group, an obvious increase of M1 macrophages from 16.3% to 30.2% and a remarkable decrease of M2 macrophages from 63.1% to 49.4% were observed in CuPP group, indicating the significantly ameliorative tumor immune microenvironment. Especially, after exposure to 1064 nm laser, much stronger polarization of macrophages from M2 phenotype to M1 phenotype was achieved, illustrating hyperthermia‐enhanced immune regulation effect. After combining with immune checkpoint blocker *α*PD‐L1, CuPP + L + *α*PD‐L1 treated mice showed the maximum distributed proportion of M1 macrophages (49.6%) and simultaneously minimum distributed proportion of M2 macrophages (28.9%) in tumor. With the reversion of immunosuppressive TME, the body's immune responses would be specifically activated and further amplified with the aid of *α*PD‐L1. As expected, CuPP + L + *α*PD‐L1 treatment could remarkably elevate the ratio of CD4^+^ and CD8^+^ T cells, which was 5.71‐ and 7.77‐fold separately relative to PBS treatment but reduce the ratio of Treg cells 3.10‐fold in tumors (**Figure** **6**A–C). Moreover, the increased secretions of IL‐12, IL‐1*β*, and TNF‐*α* but declined secretion of IL‐10 in lymph nodes could be found in CuPP + L + *α*PD‐L1 group (Figure 6D–G). All these results suggested that the synergistic treatments could induce a more supportive immune microenvironment and evoke strong immune responses.

For further implementation of translation from bench to clinical application, it is necessary to comprehensively evaluate the toxicity of nanoenzymes in vivo. At 16 days post‐injection, the hematological and serum biochemical examinations were carried out. The results exhibited that the complete blood count as well as liver and kidney function indicators were within the normal ranges, suggesting the excellent biosafety of CuPP for in vivo application (Figure 6H,I). Moreover, no distinct pathological lesions of organs could be observed in all treatment groups, reconfirming the good biocompatibility and biosafe of CuPP (Figure S26, Supporting Information).

## 3 Conclusion

In summary, for the first time, Cu‐doped PPy nanozymes containing mixed valance states (Cu^I^/Cu^II^) were successfully constructed by a straightforward one‐step procedure using CuCl_2_ as oxidizing catalysts, which possessed trienzyme‐like activities, including CAT, POD, and GPx, to specifically promote O_2_ and ·OH elevation but GSH reduction in TME, thus causing irreversible oxidative stress damage to tumor cells and reversing the redox balance. Based on the excellent photothermal conversion ability of CuPP, the local temperature elevation at the tumor site after laser irradiation further strengthened the redox hemostasis disruption by enhancing trienzyme‐like activities, which in turn initiated the reversion of the intrinsic immunosuppressive TME through re‐educating macrophage from pro‐tumoral M2 to anti‐tumoral M1 phenotype. By further combining with *α*PD‐L1, CuPP nanozyme‐based synergistic therapy could simultaneously induce potent hyperthermia, severe oxidative stress, and intense immune effect, which resulted in almost complete ablation of tumors with negligible systemic toxicity in vitro and in vivo. This work not only sheds light on a novel nanozyme based on CuPP with outstanding trienzyme‐mimic activities and immunosuppressive TME‐reversing properties but also paves an avenue toward broadening the bioapplications of PPy into enzyme‐catalytic nanomedicine.

## 4 Experimental Section

### Preparation of CuP with Different Sizes

Copper‐doped CuP nanozymes were successfully synthesized according to the authors’ previous work but using CuCl_2_ instead of FeCl_3_ as catalyst. First, various amounts of PVA (10, 30, 50, and 100 mg) were mixed with 10 mL of deionized water and then heated at 90 °C under gentle stirring for 1 h. Afterward, the above mixture cooled naturally to room temperature and the freshly prepared CuCl_2_ aqueous solution (1 g, 10 mL) was slowly added and stirred for 1 h. Subsequently, 200 µL of pyrrole monomer was added dropwise and the mixture was stirred for another 18 h at room temperature. The resulting CuP with different sizes (denoted as CuP_(10)_, CuP_(30)_, CuP_(50)_, and CuP_(100)_) were collected by centrifugating and washing with deionized water repeatedly, and finally dispersed in deionized water for future use.

### PEGylation on the Surface of CuP

To obtain PEGylated CuP (termed as CuPP), 10 mg of CuP_(30)_ were dispersed in 10 mL of deionized water and further mixed with 100 mg of DSPE‐PEG2000 (pre‐dissolving in 1 mL of liquid formed by 0.4 mL of acetone and 0.6 mL of ethanol) under ultrasound treatment in the ice bath for 30 min and then stirred for 24 h. After that, the above dispersion was centrifuged and washed with H_2_O to purify CuPP. The obtained CuPP were stored at 4 °C for further use.

### Photothermal Conversion Performance of CuPP

To systematically evaluate the photothermal conversion performance of CuPP, the increase in temperature of CuPP was monitored by exposing CuPP aqueous solutions with different concentrations (0, 5, 10, 20, and 40 µg mL^–1^) to 808 or 1064 nm laser at different power densities (0.5, 1.0, 1.5, and 2.0 W cm^–2^) for 5 min using an IR thermal camera (TI100 Infrared Camera FLK‐TI100 9HZ, FLUKE). Deionized water was irradiated as a control. To study the photothermal stability, CuPP aqueous solution (10 µg mL^–1^) was irradiated by an 808 or 1064 nm laser at the same power density of 1.0 W cm^–2^ for five repeated cycles of 6 min irradiation ON and 9 min OFF.

### Glutathione Peroxidase‐Like Activity of CuPP

The consumption of GSH over time was monitored by UV–vis spectroscopy. CuPP with various concentrations (0, 1.25, 2.5, and 5 µg mL^–1^) were mixed with GSH (1 mm) solutions under different temperatures (room temperature, 37 °C, and 50 °C). At different time points, the solution was taken out and mixed with 5,5′‐dithiobis‐(2‐nitrobenzoic acid) (DTNB) (10 mg mL^–1^), and then the UV–vis spectroscopy was applied to detect the absorbance of the above suspension.

### Peroxidase‐Like Activity of CuPP

The ability of CuPP to catalyze H_2_O_2_ to generate ·OH was determined by mixing CuPP (0, 10, 20, and 40 µg mL^–1^) with MB (10 µg mL^–1^) and H_2_O_2_ (10 mm) at different temperatures (room temperature, 37 °C, and 50 °C). The absorbance of MB at 664 nm was recorded by UV–vis spectroscopy. Besides, the generation of ·OH caused by different treatments (Control, L, CuPP, and CuPP + L) at room temperature were also measured. Then, ·OH generation between CuPP and FePP under the same [Fe][Cu] molar mass concentration (0.016 mm) at different pH were further evaluated. To confirm the generation of ·OH, electron spin resonance (ESR) spectra were also measured on a Bruker Model A300 spectrometer using 5,5‐dimethyl‐1‐pyrrolineN‐oxide (DMPO) as the ·OH trapping agent.

### Catalase‐Like Activity of CuPP

The dissolved O_2_ concentrations of CuPP in the presence of H_2_O_2_ were measured by a JPSJ‐605F portable dissolved oxygen meter (Leici Instrument Co., Ltd., Shanghai, China). Briefly, CuPP dispersed in PBS (pH 7.4) with different concentrations (0, 10, 20, and 40 µg mL^–1^) were filled with argon and sealed with parafilm. Then the real‐time O_2_ concentrations were recorded at different temperatures (room temperature, 37 °C, and 50 °C) after injection of H_2_O_2_ (10 mm). Besides, the generation of O_2_ caused by different treatments (Control, L, CuPP, and CuPP + L) at room temperature were also measured.

### Cellular Uptake

To investigate the cellular internalization profile of CuPP, 4T1 cells were incubated with Cy5‐labeled CuPP (Cy5 concentration: 5 µg mL^–1^) for different times (0.5, 1, 2, 4, and 8 h) at 37 °C. At the specific time point, the redundant media were removed by washing with PBS for three times. Afterward, the cells were fixed with 4% paraformaldehyde solution and stained by DAPI, followed by observation under confocal laser scanning microscopy (CLSM, TCS SP5II, Leica, Germany).

### Cytotoxicity of CuPP

Both cancer cell lines (U87 and 4T1 cells) and healthy cell lines (HUVEC and BV2 cells) were seeded in 96‐well plates at the density of 8 × 10^3^ cells per well and cultured overnight. Then, different concentrations (0, 2.5, 5, 10, 20, 40, 80, and 120 µg mL^–1^) of CuPP were added to replace the medium. After incubated for 24 or 48 h, the cell viability assay was conducted following the standard protocol to detect the relative cell viability by using the SpectraMax M2 plate reader (Molecular Devices, CA, USA).

To evaluate in vitro photo‐enhanced cytotoxicity of CuPP, 4T1 cancer cells (8 × 10^3^ cells/well) were co‐cultured with CuPP with various concentrations (0, 2.5, 5, 10, 20, and 40 µg mL^–1^) for 6 h. Afterward, the cells were irradiated with 1064 nm laser (1.0 W cm^–2^) for 5 min and incubated for another 24 h. Finally, cell viabilities were determined by CCK‐8 assay.

### Intracellular O_2_ and ROS Generation, as well as GSH Depletion

For O_2_ generation, 4T1 cells were seeded in the confocal dish with a density of 2 × 10^5^ cells to adhere and then cultured in a simulated hypoxic atmosphere for 8 h by using Anaerobic gas bag (5% anaerobic, MGC). Following, these cells were incubated with [Ru(dpp)_3_]Cl_2_ (5 µm) for 2 h, followed by rinsing with PBS three times to remove free [Ru(dpp)_3_]Cl_2_. Then, the medium was replaced with pure DMEM or CuPP‐contained DMEM (20 µg mL^–1^) for 8 h and then the cells in L group and CuPP + L group were irradiated by a 1064 nm laser (1 W cm^–2^) for 5 min. All the images were acquired under CLSM.

4T1 cells were seeded in the confocal dish with a density of 2 × 10^5^ cells to adhere and then co‐cultured with different formulations (CuPP concentration: 40 µg mL^–1^) for 6 h. The cells in laser treatment groups were exposed to a 1064 nm laser (1 W cm^–2^) for 5 min. For intracellular ROS generation, the cells were stained with DCFH‐DA (20 µm) for 30 min. For intracellular GSH depletion, the cells were stained with ThiolTracker Violet (20 µm) for 30 min. All the images were acquired under CLSM.

### In Vitro Macrophage Polarization

RAW 264.7 macrophages were cultured with IL‐4 (25 ng mL^–1^) for 12 h to induce M2 polarization. The supernatant of 4T1 cells with untreated, 1064 nm only, CuPP (40 µg mL^–1^) with or without 1064 nm (1 W cm^–2^, 5 min) laser irradiation were used to incubate with M2 macrophages for another 12 h. Afterward, RAW 264.7 macrophages were collected and stained by PE anti‐CD86 and FITC anti‐CD206 antibodies, and then were measured by flow cytometry (Guava EasyCyte). The level of cytokines (IL‐12 and IL‐10) in supernatants was collected and detected through ELISA assay.

### Animal Model

Female Balb/c mice (16–18 g, 5–8 weeks old) were purchased from the Laboratory Animal Center of Sun Yat‐sen University (Guangzhou, China). All animal experiments were performed according to the guidelines approved by the Institutional Animal Care and Use Committee of Sun Yat‐sen University (Approval number: SYSU‐IACUC‐2021‐000596). 2 × 10^6^ 4T1 cells were subcutaneously injected into the right back of Balb/c mice. When the tumor became distinct and the tumor volume reached ≈80 mm^3^, the mice were randomly assigned into either control or test groups.

### In Vivo Biological Distribution

The mice bearing 4T1 tumor were used for in vivo biological distribution study of Cy5‐labelled CuPP. After intravenous injection of Cy5‐labelled CuPP (2.5 mg kg^–1^ Cy5 per mouse), the mice were imaged by the In‐Vivo Imaging System (Bruker, FX Pro, USA) at different times (2, 4, 8, 12, and 24 h). Then, the main organs (heart, liver, spleen, lung, and kidney) and tumors were collected and imaged immediately.

### In Vivo Photoacoustic Imaging

For in vivo photoacoustic (PA) imaging, the mice bearing 4T1 tumor were administrated with CuPP at a dose of 20 mg kg^–1^ via intravenous injection. PA images of the tumor site and quantitative analysis of PA signal intensities were performed at different time points (0, 2, 4, 8, 12, and 24 h) and the signs were recorded by a PA instrument (TomoWave Laboratories, LOIS‐3D, USA) with 1064 nm wavelength. The ImageJ was used to analyze PA signals in each region of interest.

### In Vivo Antitumor Therapy and Antitumor Immunity

To evaluate the in vivo antitumor effect of CuPP, the mice bearing 4T1 tumor were randomly divided into six groups (*n* = 5): 1) Control group, 2) L group, 3) *α*PD‐L1 group, 4) CuPP group, 5) CuPP + L group, and 6) CuPP + L + *α*PD‐L1 group. Mice were intravenously injected with PBS in group (1), (2), and (3), while with CuPP dispersed in PBS at the dosage of 20 mg kg^–1^ for group (4), (5), and (6). At 12 h post‐injection, the tumor sites of group (2), (5), and (6) received a 1064 nm laser irradiation (1 W cm^–2^) for 5 min. Meanwhile, an IR thermal camera was used to monitor the local temperature of tumors and to collect IR thermal images of the whole mice body. At day 1, 4, and 7, 50 µg of *α*PD‐L1 in 100 µL PBS was injected intraperitoneally for group (3) and (6). The body weight and tumor volume of each mouse were measured every other day and the tumor volume was calculated using the formula: Tumor volume = 1/2 × (tumor width)^2^ × (tumor length). 16 days later, the mice were sacrificed to collect main tissues (heart, liver, spleen, lung, kidney, and tumor) for histological analysis.

For macrophage polarization analysis, the tumors of mice were collected, homogenized in PBS, and filtered to gain single‐cell suspension. Then, the cell suspension of tumors were stained with PE anti‐CD11b, FITC anti‐CD86 or PE anti‐CD11b, FITC anti‐CD206 antibodies for macrophage phenotype analysis by flow cytometry, respectively. At the same time, the cell suspensions of tumors were stained by APC anti‐CD3, PE anti‐CD8a, and FITC anti‐CD4 for T cells activation analysis by flow cytometry, and cells were stained by APC anti‐CD3, FITC anti‐CD4, and PE anti‐Foxp3 to detect Tregs. Besides, the lymph nodes of mice were gathered. The contents of cytokines (TNF‐*α*, IL‐10, IL‐1*β*, and IL‐12) were measured using ELISA kits.

The toxicology analysis was implemented by intravenously injecting diverse concentrations (0, 10, 20, and 30 mg kg^–1^) of CuPP into the Balb/c mice (*n* = 3). At day 16 after intravenous injection, the blood of these mice were gathered for biochemistry assay.

### Statistical Analysis

All statistical analyses were used GraphPad Prism. The results of statistical analysis were presented as mean ± SD. Statistical significance was calculated by one‐way ANOVA analysis. The statistical significance was defined as *p < 0.05; **p < 0.01; ***p < 0.001.

## Conflict of Interest

The authors declare no conflict of interest.

## Supporting information

Supporting Infor

## Data Availability

The data that support the findings of this study are available from the corresponding author upon reasonable request.
