# Supplementary material for: Polypyrrole Nanoenzymes as Tumor Microenvironment Modulators to Reprogram Macrophage and Potentiate Immunotherapy
Source: Adv Sci (Weinh). 2022 Jun 9;9(23):2201703. doi: 10.1002/advs.202201703 (PMC9376744; doi:10.1002/advs.202201703)
Supplement: Supplementary file 1 — Supporting Infor [file ADVS-9-2201703-s001.pdf]

ADVANCED SCIENCE
Open Access

Supporting Information

for *Adv. Sci.*, DOI 10.1002/advs.202201703

Polypyrrole Nanoenzymes as Tumor Microenvironment Modulators to Reprogram Macrophage and Potentiate Immunotherapy

Weiwei Zeng, Mian Yu, Ting Chen, Yuanqi Liu, Yunfei Yi, Chenyi Huang, Jia Tang, Hanyue Li, Meitong Ou, Tianqi Wang, Meiying Wu and Lin Mei**

© Copyright 2022. WILEY-VCH GmbH.

Supporting Information

Polypyrrole Nanoenzymes as Tumor Microenvironment Modulators to Reprogram Macrophage and Potentiate Immunotherapy

Weiwei Zeng, Mian Yu, Ting Chen, Yuanqi Liu, Yunfei Yi, Chenyi Huang, Jia Tang, Hanyue Li, Meitong Ou, Tianqi Wang, Meiying Wu, Lin Mei**

Supporting Information for

Polypyrrole Nanoenzymes as Tumor Microenvironment Modulators to Reprogram Macrophage and Potentiate Immunotherapy

Weiwei Zeng [a, b], *Mian Yu* [a], *Ting Chen* [a], *Yuanqi Liu* [a], *Yunfei Yi* [a], *Chenyi Huang* [a], *Jia Tang* [a], *Hanyue Li* [b], *Meitong Ou* [a], *Tianqi Wang* [a], *Meiying Wu* [a,*], *Lin Mei* [a,b,*]

[a] School of Pharmaceutical Sciences (Shenzhen), Shenzhen Campus of Sun Yat-sen University, Shenzhen 518107, China

[b] Tianjin Key Laboratory of Biomedical Materials, Key Laboratory of Biomaterials and Nanotechnology for Cancer Immunotherapy, Institute of Biomedical Engineering, Chinese Academy of Medical Sciences & Peking Union Medical College, Tianjin 300192, China

* Corresponding authors.

E-mail: wumy53@mail.sysu.edu.cn (M. Wu), meilin7@mail.sysu.edu.cn (L. Mei)

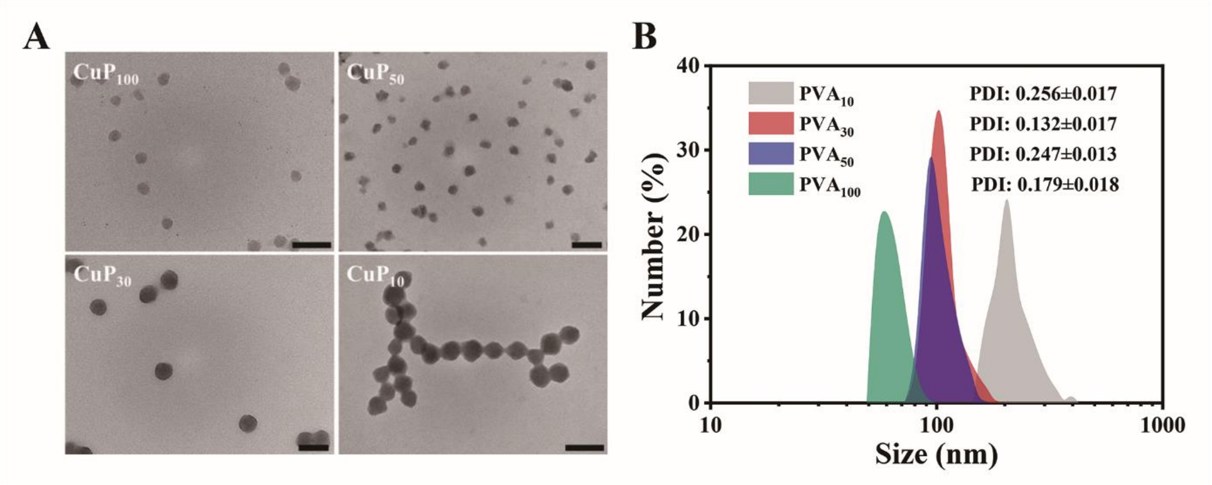

Figure S1. (A) TEM images and (B) corresponding size distributions of CuP with different adding amounts of PVA (10, 30, 50, and 100 mg). Scale bar: 100 nm.

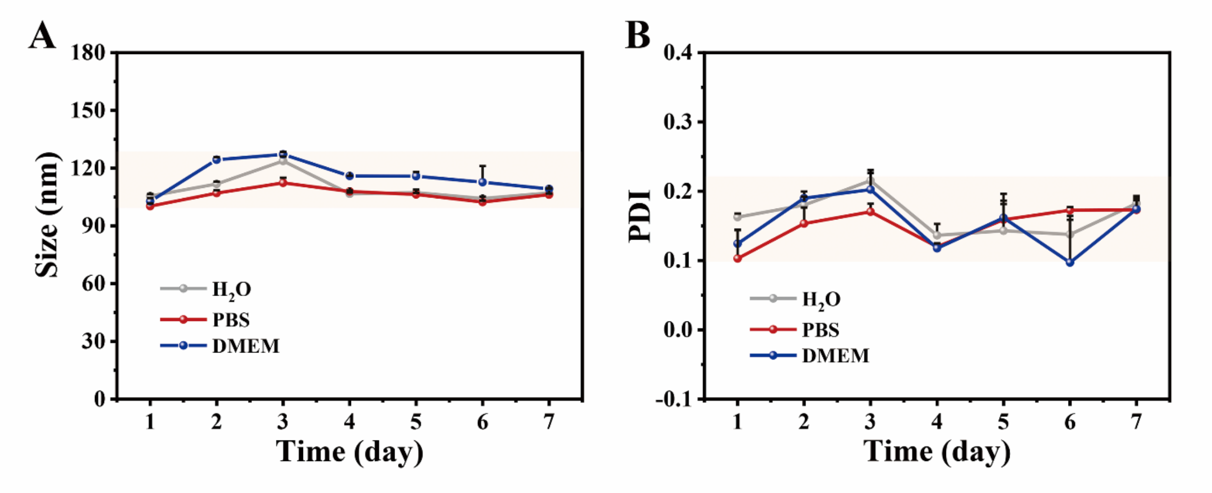

Figure S2. (A) Hydrodynamic diameter changes and (B) corresponding PDI changes of CuPP dispersed in different physiological media at varied time points. Data represent means ± SD (n = 3).

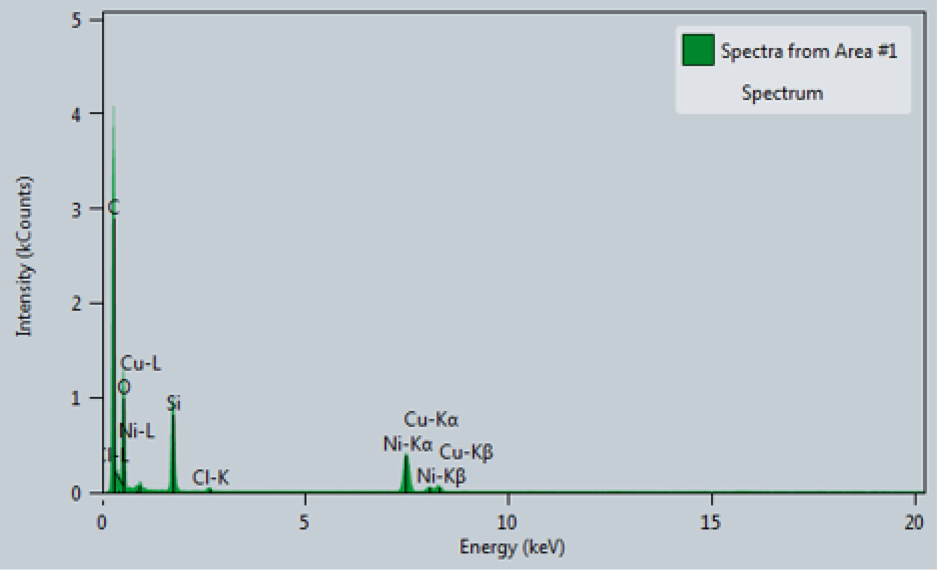

Figure S3. X-ray energy dispersive spectroscopy (EDS) of CuP.

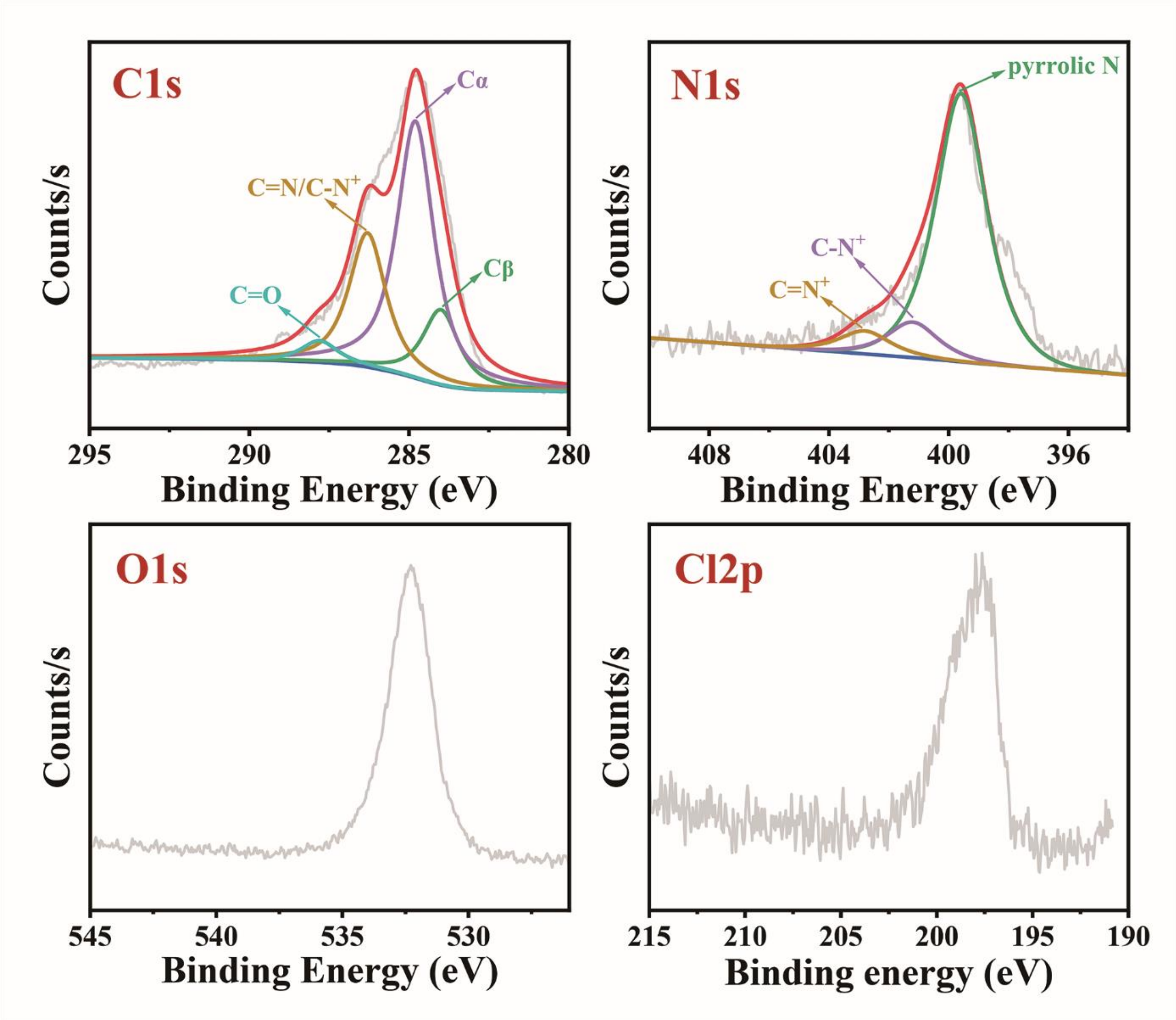

Figure S4. High-resolution C1s, N1s, O1s, and Cl2p XPS spectra of CuP.

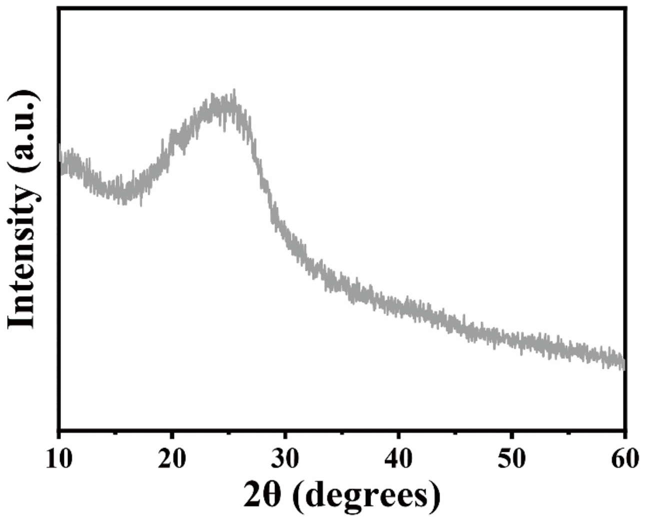

Figure S5. XRD pattern of CuP.

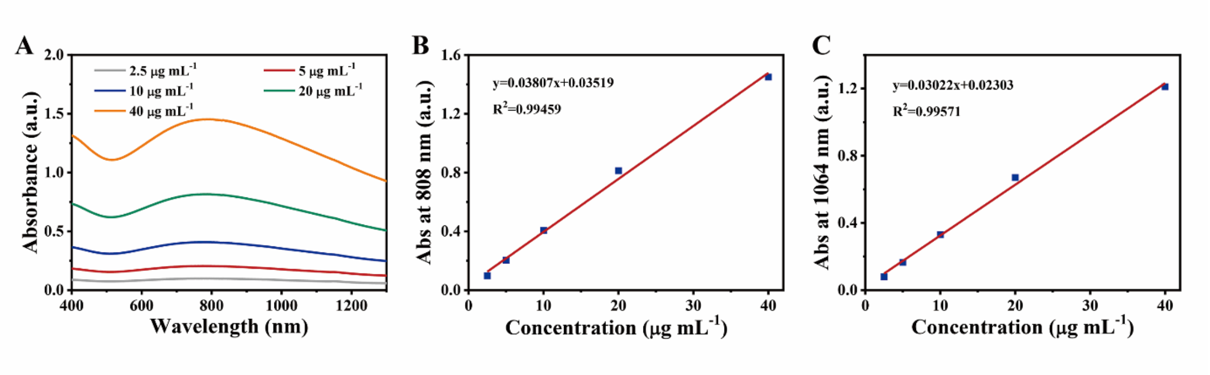

Figure S6. (A) UV-vis-NIR spectra of various concentrations of CuPP. Mass extinction coefficient (ε) of CuPP at (B) 808 and (C) 1064 nm laser irradiation.

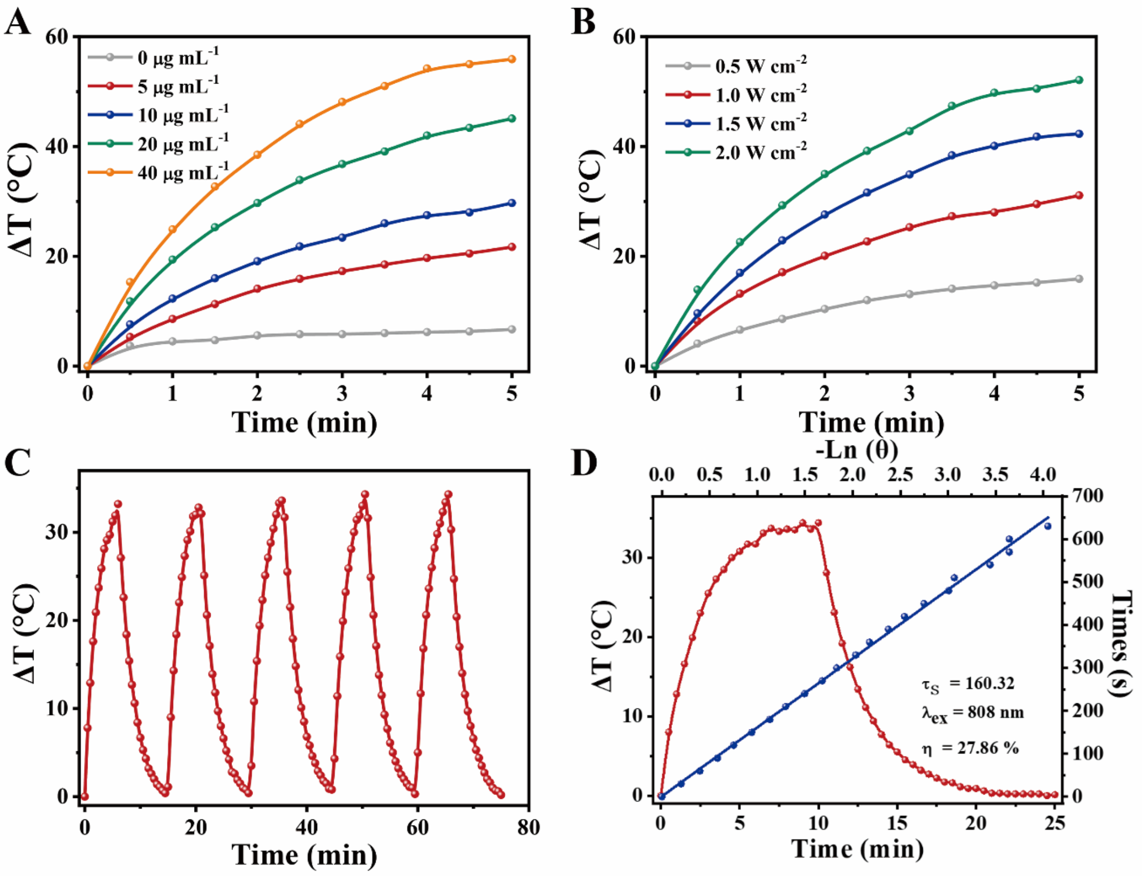

Figure S7. (A) Temperature change profiles of CuPP with different concentrations under 808 nm laser irradiation for 5 min at the power density of 1.0 W cm^{-2}. (B) Temperature change profiles of CuPP (10 μg mL^{-1}) exposed to different power densities (0.5-2.0 W cm^{-2}) of 808 nm laser. (C) Photothermal conversion stability of CuPP aqueous solution for five laser on/off cycles under the irradiation of 808 nm laser. (D) Photothermal heating and cooling curves of CuPP under 808 nm laser irradiation and corresponding linear relationship between time and -lnθ from the cooling period.

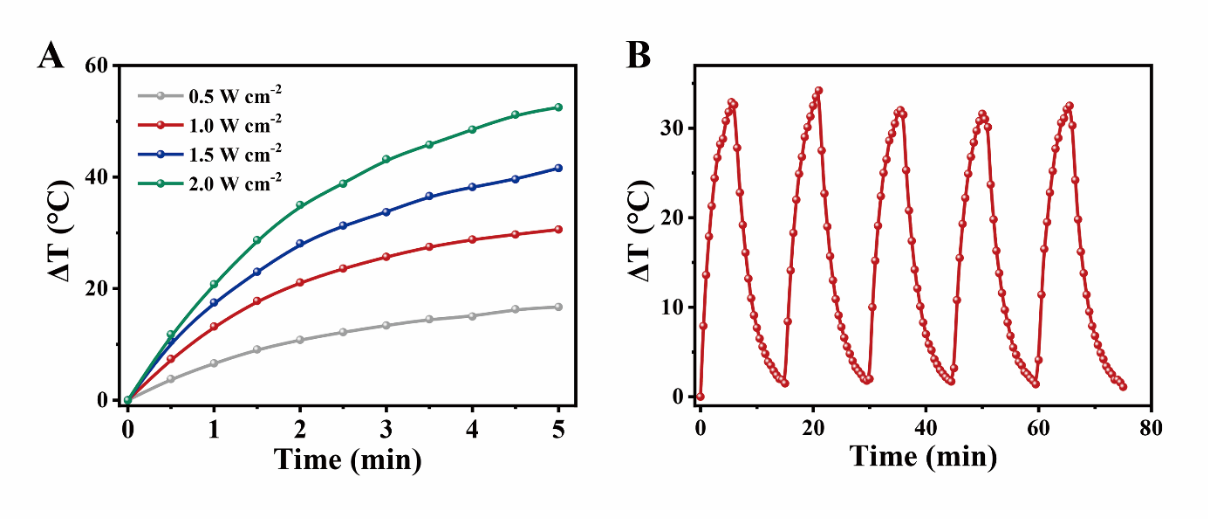

Figure S8. (A) Temperature change profiles of CuPP (10 μg mL^{-1}) exposed to different power densities (0.5-2.0 W cm^{-2}) of 1064 nm laser. (B) Photothermal conversion stability of CuPP aqueous solution for five laser on/off cycles under the irradiation of 1064 nm laser.

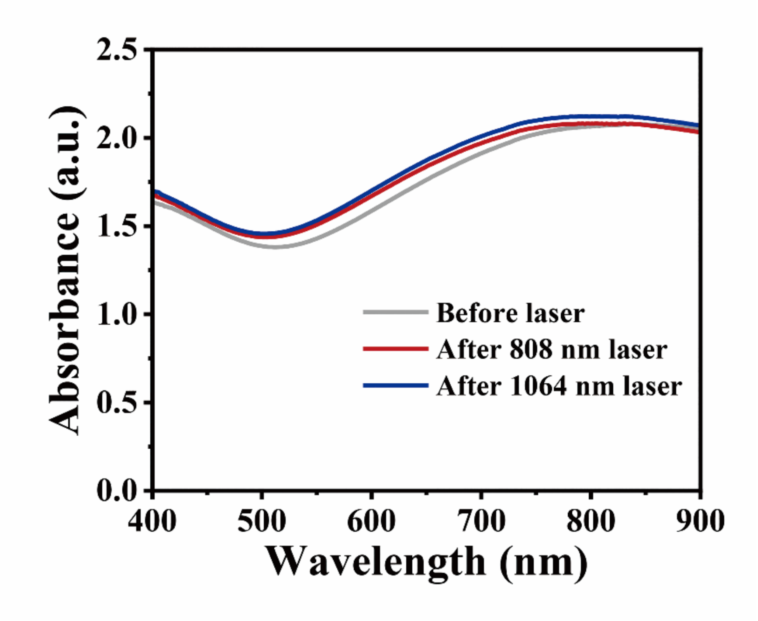

Figure S9. UV-vis-NIR absorbance spectra of CuPP aqueous dispersions before and after 808 or 1064 nm laser irradiation for 30 min.

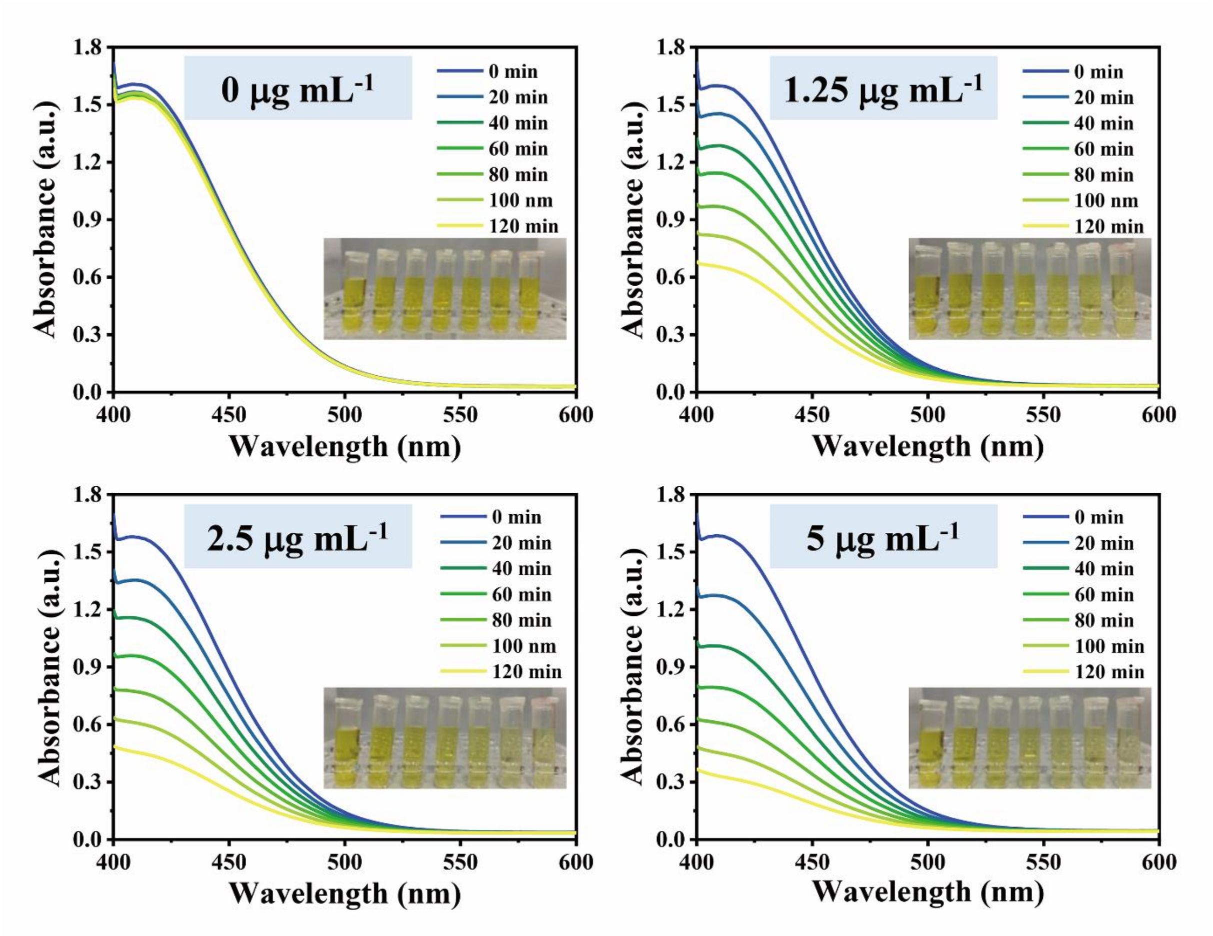

Figure S10. GSH depletion profile treated with CuPP at different concentrations (0, 1.25, 2.5, and 5 μg mL^{-1}) under room temperature. Inset: the photographs of the solutions at different time points.

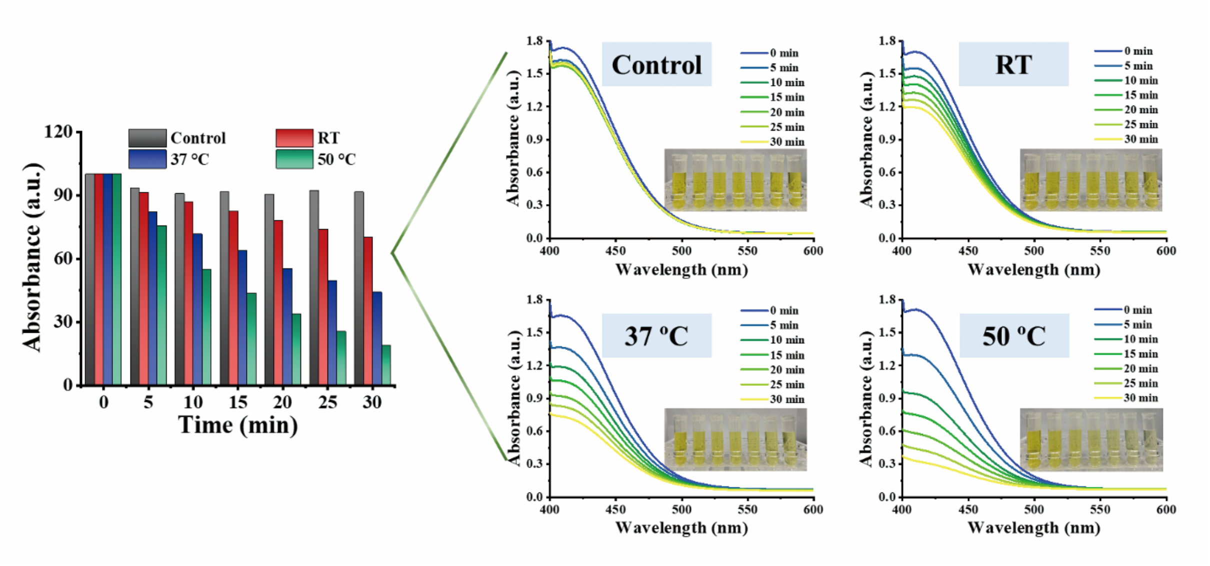

Figure S11. GSH depletion profile treated with or without CuPP (5 μg mL^{-1}) at different temperature (RT, 37 °C, and 50 °C). Inset: the photographs of the solutions at different time points.

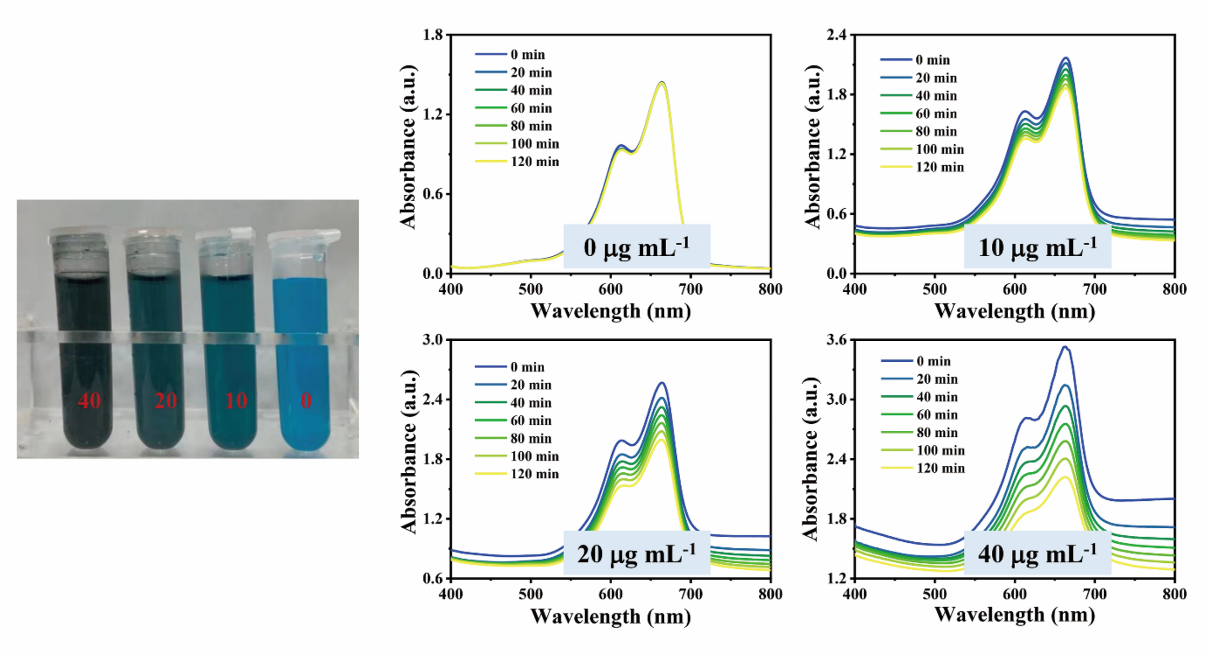

Figure S12. MB degradation profile treated with CuPP at different concentrations (0, 10, 20, and 40 μg mL^{-1}) under room temperature. And the photograph is the solutions at experimental destination.

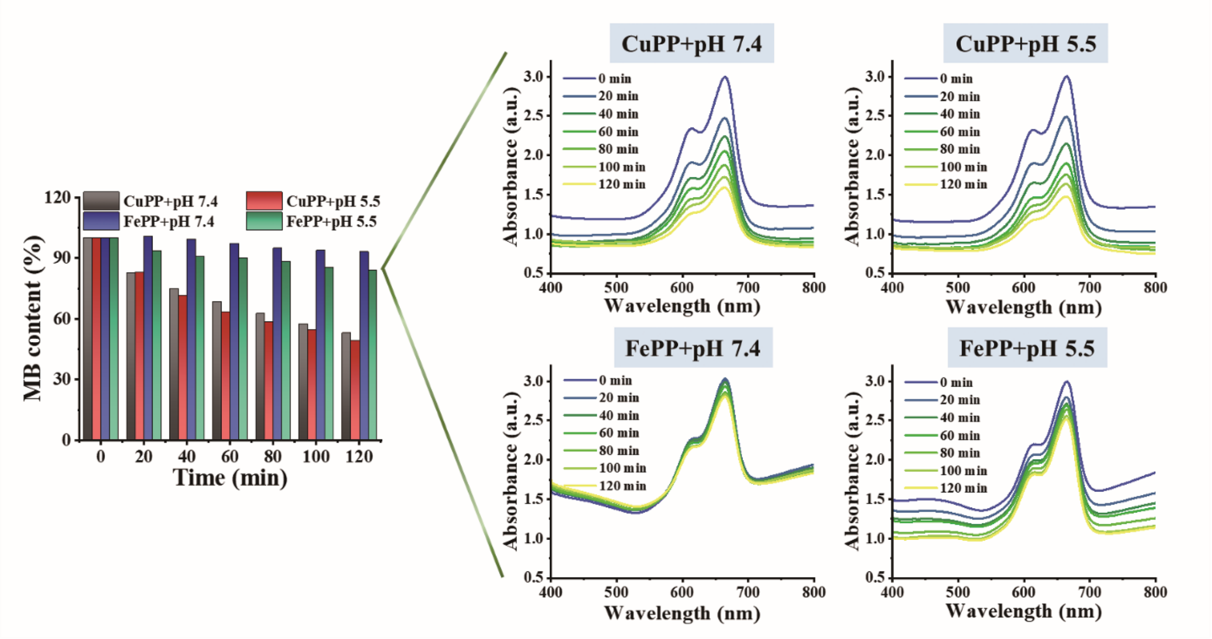

Figure S13. MB degradation profile treated with CuPP or Fe-doped polypyrrole (FePP) under the same [Fe][Cu] molar mass concentration at different pH.

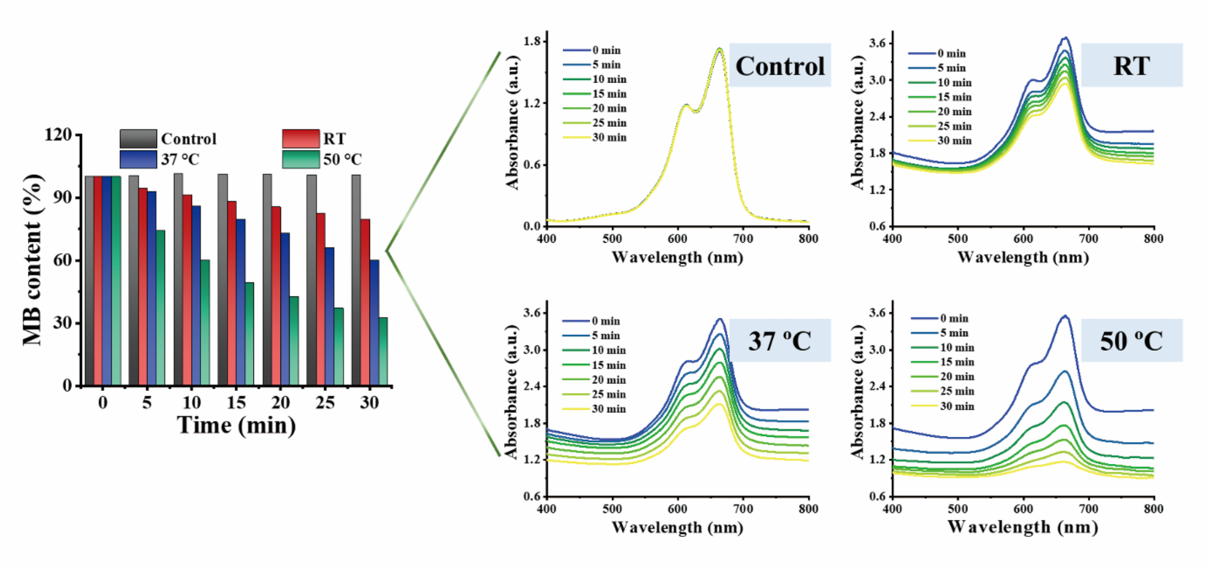

Figure S14. MB degradation profile treated with or without CuPP (40 μg mL^{-1}) at different temperature (RT, 37 °C, and 50 °C).

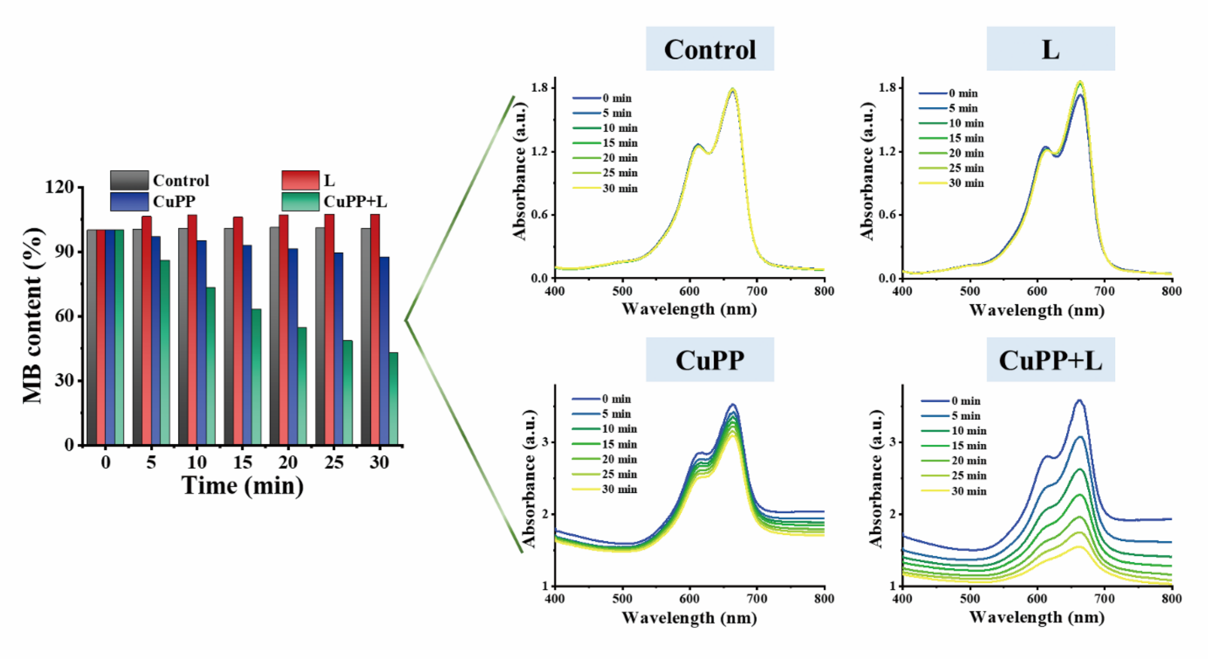

Figure S15. MB degradation profile after different treatments (Control, L, CuPP, and CuPP+L) at room temperature.

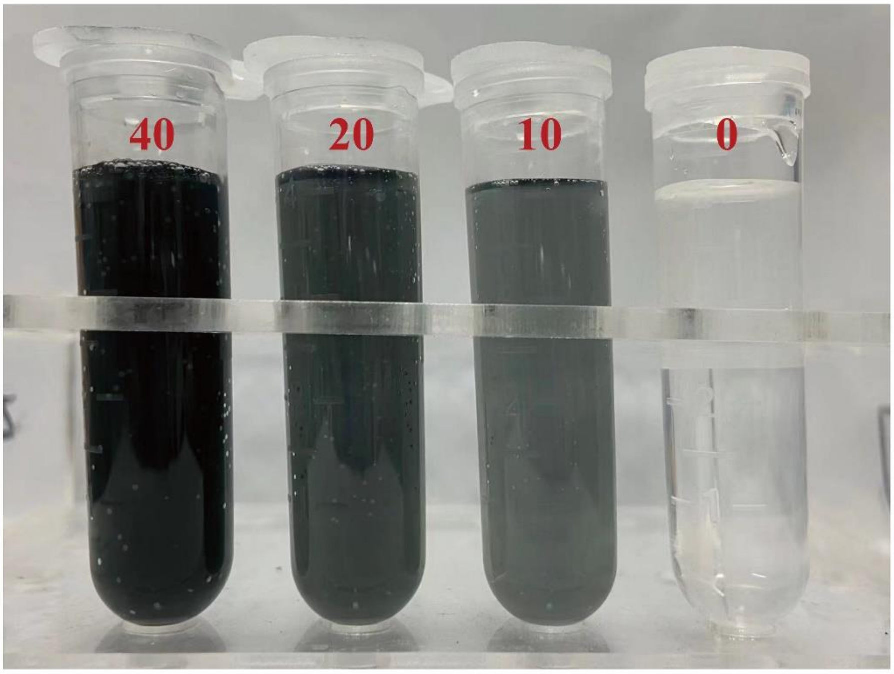

Figure S16. The photograph of O_2 generation in H_2O_2 solutions containing different concentrations of CuPP.

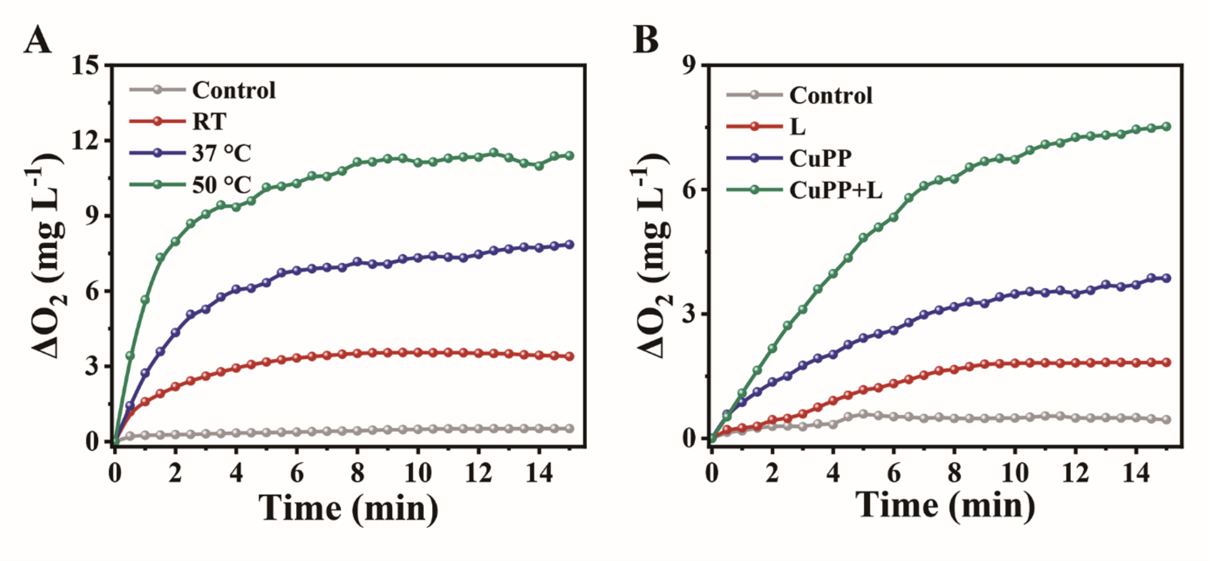

Figure S17. (A) Dissolved O_2 profile at different temperature (RT, 37 °C, and 50 °C). (B) Dissolved O_2 profile after different treatments (Control, L, CuPP, and CuPP+L) at room temperature.

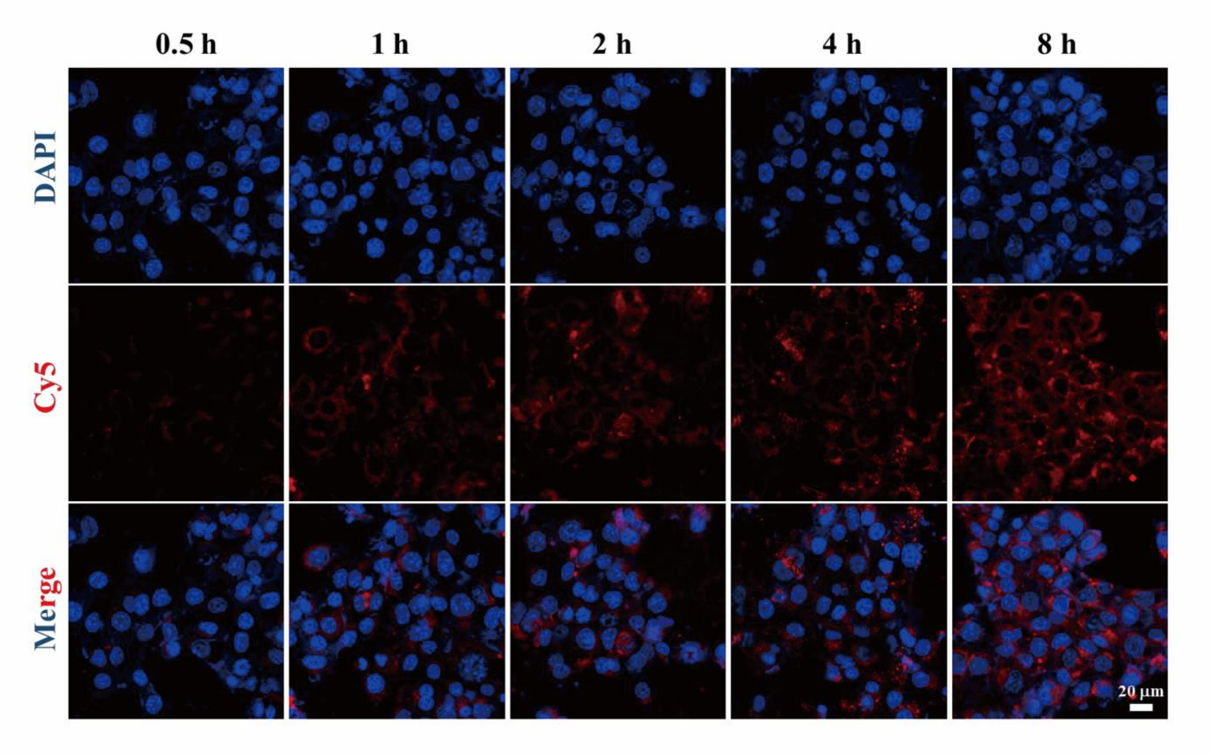

Figure S18. CLSM images of 4T1 cells incubated with Cy5-labelled CuPP for different times (0.5, 1, 2, 4, and 8 h). Blue and red colors represent DAPI and Cy5 fluorescence, respectively. Images share the same scale bar (20 µm).

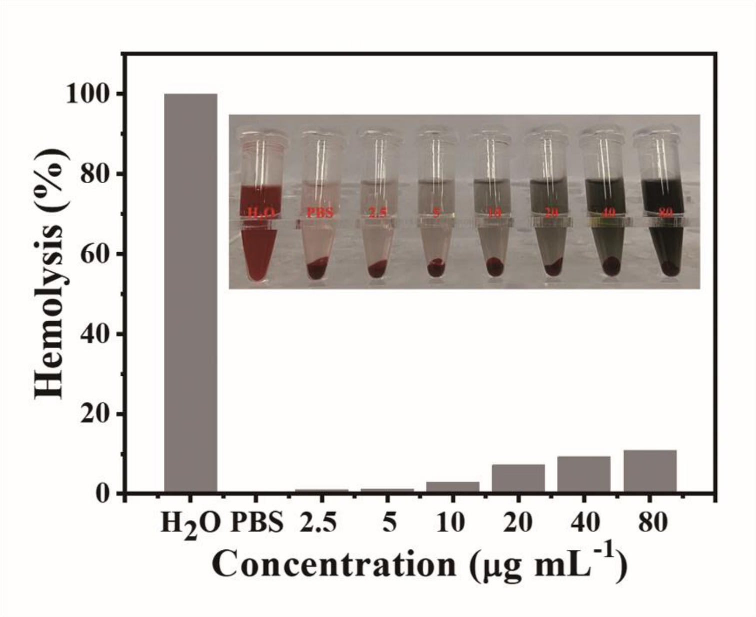

Figure S19. Hemolysis assay of red blood cells treated with water, PBS and CuPP dispersed in PBS at different concentrations.

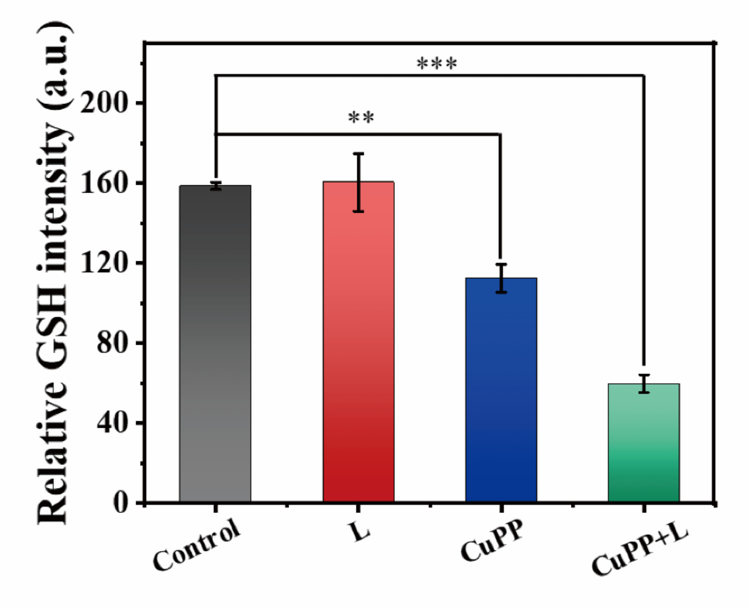

Figure S20. The quantification of intracellular GSH depletion in 4T1 cells after different treatments. Data represent means ± SD (n = 3). Statistical significance was calculated by one-way ANOVA analysis. *P < 0.05; **P < 0.01; ***P < 0.001.

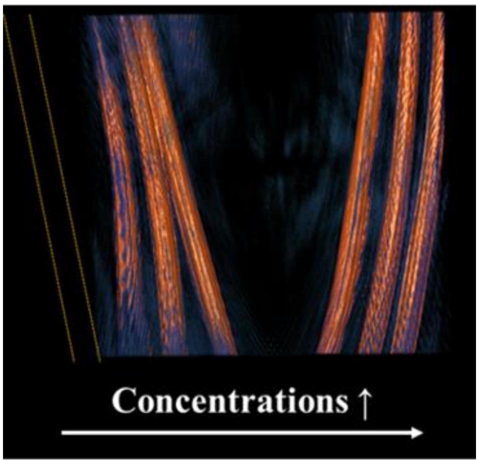

Figure S21. *In vitro* PA imaging of CuPP with various concentrations (0, 0.0625, 0.125, 0.25, 0.5, 1.0, and 2.0 mg mL^{-1}) under 1064 nm laser irradiation.

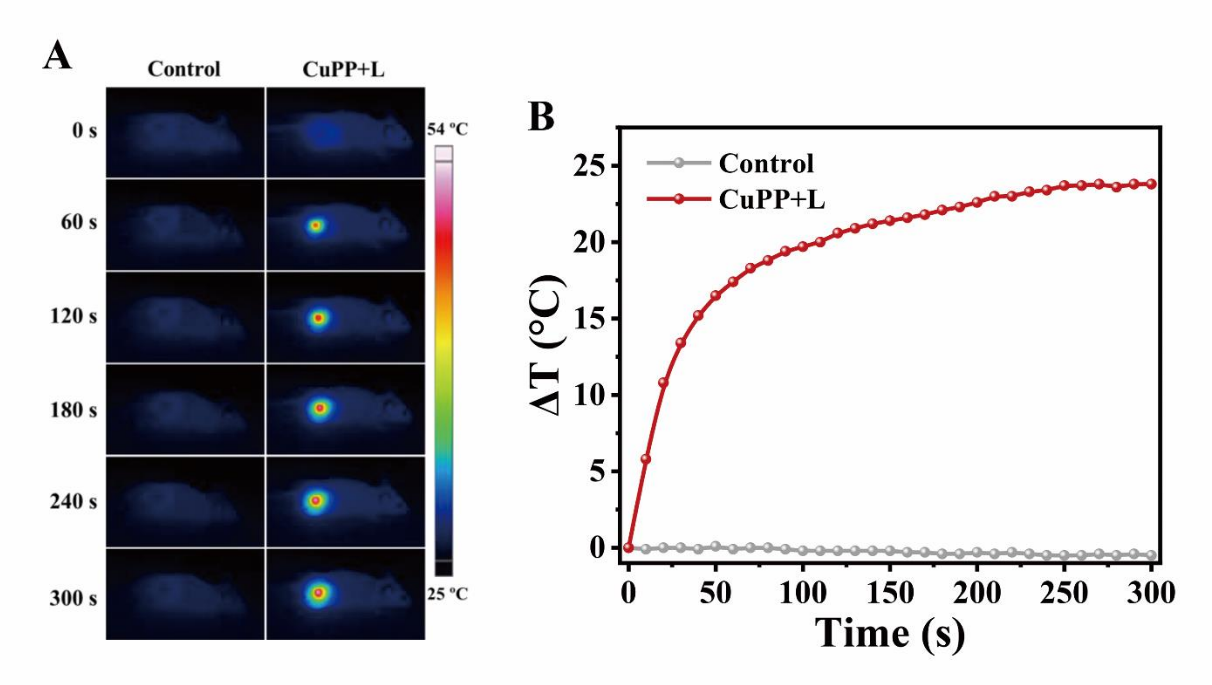

Figure S22. (A) IR thermal images of tumor-bearing mice in different groups during 1064 nm laser irradiation (1.0 W cm^{-2}) taken at different time intervals. (B) The corresponding temperature elevation at the tumor site with different treatments.

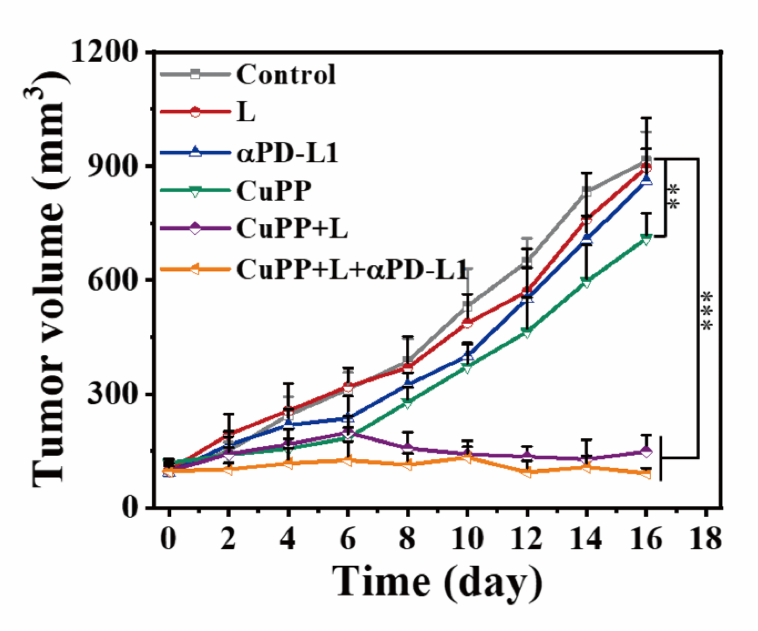

Figure S23. Relative tumor volumes of mice in different groups. Data represent means ± SD (n = 5). Statistical significance was calculated by one-way ANOVA analysis. *P < 0.05; **P < 0.01; ***P < 0.001.

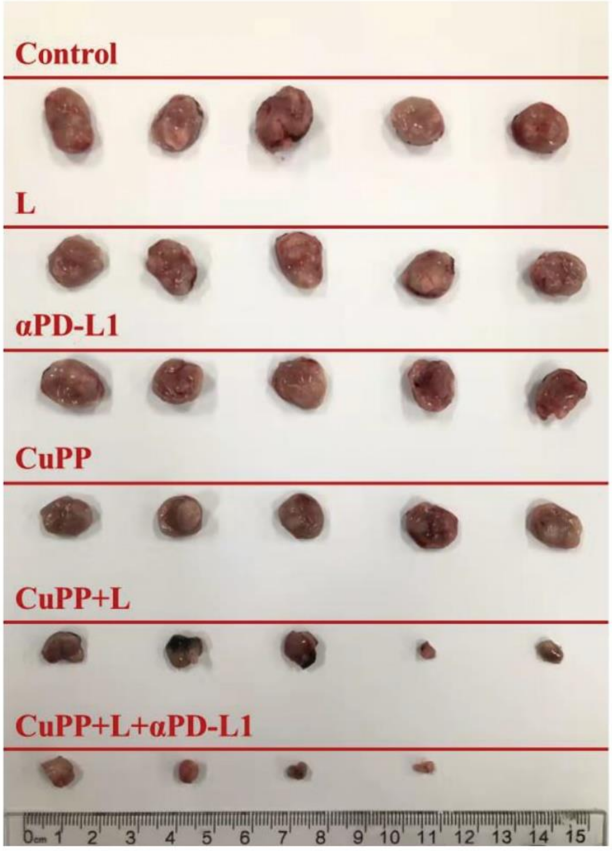

Figure S24. Photographs of excised tumor tissues at day16 after different treatments.

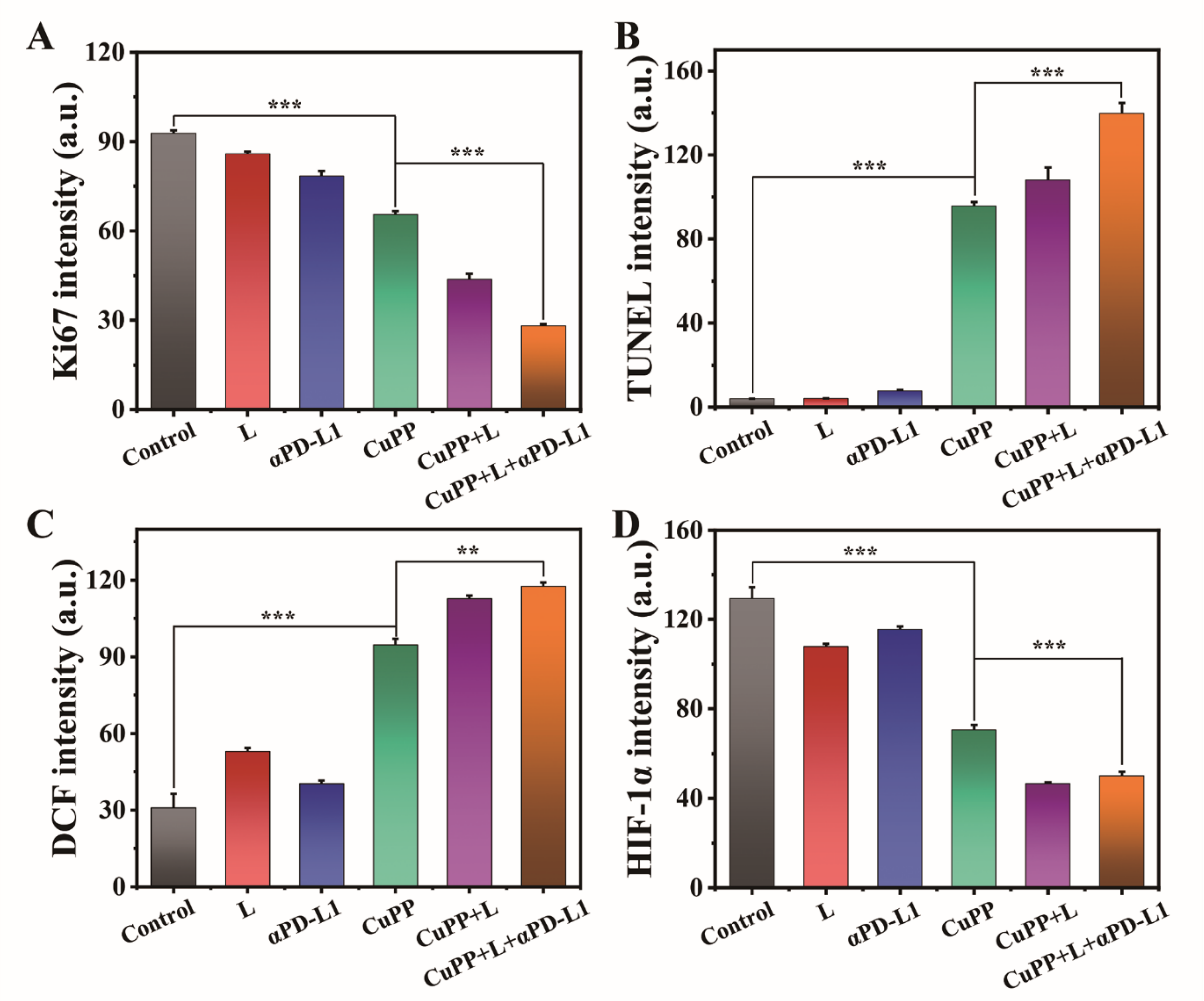

Figure S25. Quantification of (A) Ki67, (B) TUNEL, (C) DCF, and (D) HIF-1α stainings of tumor tissues collected after various treatments. Data represent means ± SD (n = 3). Statistical significance was calculated by one-way ANOVA analysis. *P < 0.05; **P < 0.01; ***P < 0.001.

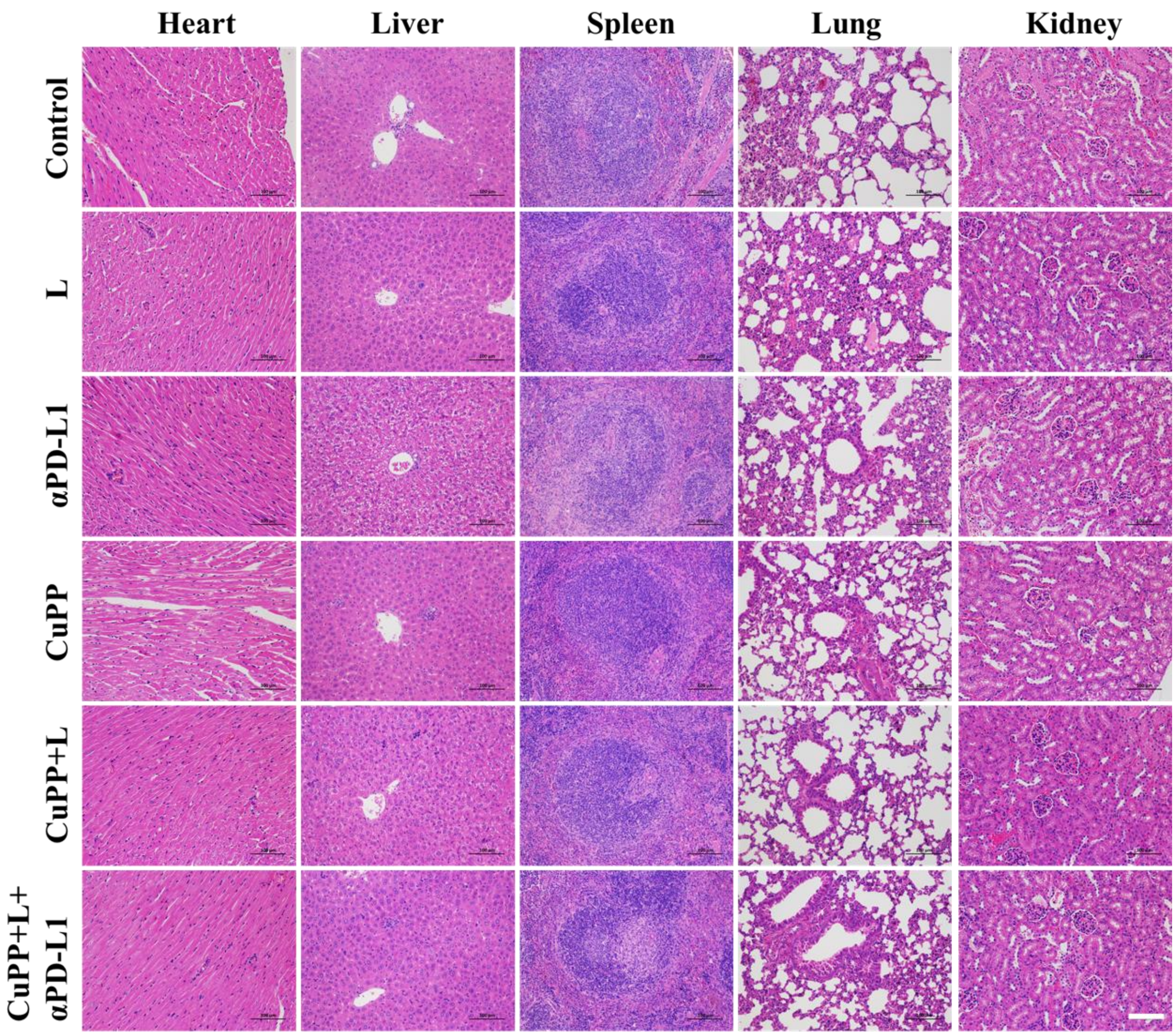

Figure S26. HE analyses of major organs (heart, liver, spleen, lung, and kidney) at the endpoint of treatments (scale bar: 100 µm).
